# CRF07_BC is associated with slow HIV disease progression in Chinese patients

**DOI:** 10.1038/s41598-022-07518-4

**Published:** 2022-03-08

**Authors:** Jingrong Ye, Jing Chen, Juan Wang, Yuncong Wang, Hui Xing, Fengting Yu, Lifeng Liu, Yang Han, Huihuang Huang, Yi Feng, Yuhua Ruan, Minna Zheng, Xinli Lu, Xiaoli Guo, Hong Yang, Qi Guo, Yi Lin, Jianjun Wu, Shouli Wu, Yilong Tang, Xiaoguang Sun, Xiaobai Zou, Guolong Yu, Jianjun Li, Quanhua Zhou, Ling Su, Lincai Zhang, Zhan Gao, Ruolei Xin, Shufang He, Conghui Xu, Mingqiang Hao, Yinxiao Hao, Xianlong Ren, Jie Li, Lishi Bai, Tianjun Jiang, Tong Zhang, Yiming Shao, Hongyan Lu

**Affiliations:** 1 Institute for HIV/AIDS and STD Prevention and Control, Beijing Center for Disease Prevention and Control (CDC) and Beijing Research Center for Preventive Medicine, No.16, Hepingli Middle Street; Dong Chen District, Beijing, China; 2 Division of Virology and Immunology, State Key Laboratory for Infectious Disease and Prevention and Control and National Center for AIDS/STD Prevention and Control (NCAIDS), China CDC, No.155 Changbai Road, Changping District, Beijing, China; 3 Clinical and Research Center of Infectious Diseases, Beijing DiTan Hospital, Capital Medical University, No. 8, Jingshun East Street, Chaoyang District, Beijing, China; 4 Center for Infectious Diseases, Beijing YouAn Hospital, Capital Medical University, No. 8 of the Xitoutiao Outside the YouAnmen, Feng Tai District, Beijing, China; 5 Department of Infectious Disease, Peking Union Medical College Hospital, No. 1, Wangfujing Shuaifuyuan, Beijing, China; 6 Treatment and Research Center for Infectious Diseases, The Fifth Medical Center of PLA General Hospital of China, No.100 Western 4th Ring Middle Road, Fengtai District, Beijing, China; 7 Institute for HIV/AIDS and STD Prevention and Control, Tianjin CDC, No. 6 Huayue Road, Hedong District, Tianjin, China; 8 Institute for HIV/AIDS and STD Prevention and Control, Hebei CDC, No. 97 Huaian East Road, Shijiazhuang, Hebei China; 9 Institute for HIV/AIDS and STD Prevention and Control, Shanxi CDC, No. 8 Xiaonanguan, Shuangta West Street, Yingze District, Taiyuan, Shanxi China; 10 Institute for HIV/AIDS and STD Prevention and Control, Inner Mongolia CDC, No. 50 Erdos Street, Hohhot, Inner Mongolia China; 11 Institute for HIV/AIDS and STD Prevention and Control, Jilin CDC, No. 3145 Jingyang Road Changchun, Jilin, China; 12 Institute for HIV/AIDS and STD Prevention and Control, Shanghai CDC, No. 1380 Zhongshan West Road, Shanghai, China; 13 Institute for HIV/AIDS and STD Prevention and Control, Anhui CDC, No. 12560 Fanhua Road, Economic and Technological Development Zone, Hefei, Anhui China; 14 Institute for HIV/AIDS and STD Prevention and Control, Fujian CDC, No. 76 Jintai Road, Gulou District, Fuzhou, Fujian China; 15 Institute for HIV/AIDS and STD Prevention and Control, Jiangxi CDC, No. 555 Beijing East Road, Nanchang, Jiangxi China; 16 Institute for HIV/AIDS and STD Prevention and Control, Shandong CDC, No. 16992 Jingshi Road, Jinan, Shandong China; 17 Institute for HIV/AIDS and STD Prevention and Control, Hunan CDC, No. 450 Furong Middle Road, 1st Section, Changsha, Hunan China; 18 Institute for HIV/AIDS and STD Prevention and Control, Guangdong CDC, No. 160 Qunxian Road, Dashi Street, Panyu District, Guangzhou, Guangdong China; 19 Institute for HIV/AIDS and STD Prevention and Control, Guangxi CDC, No. 18 Jinzhou Road Nanning, Guangxi, China; 20 Institute for HIV/AIDS and STD Prevention and Control, Chongqing CDC, No. 8 Yangtze 2nd Road, Yuzhong District, Chongqing, China; 21 Institute for HIV/AIDS and STD Prevention and Control, Sichuan CDC, No. 6 Middle School Road, Chengdu, Sichuan China; 22 Institute for HIV/AIDS and STD Prevention and Control, Gansu CDC, No. 310 Donggang West Road, Lanzhou, Gansu China; 23 Beijing Red Cross Blood Center, No. 37 North 3rd Ring Middle Road, Haidian District, Beijing, China

**Keywords:** HIV infections, Viral evolution, Viral pathogenesis

## Abstract

HIV subtypes convey important epidemiological information and possibly influence the rate of disease progression. In this study, HIV disease progression in patients infected with CRF01_AE, CRF07_BC, and subtype B was compared in the largest HIV molecular epidemiology study ever done in China. A national data set of HIV *pol* sequences was assembled by pooling sequences from public databases and the Beijing HIV laboratory network. Logistic regression was used to assess factors associated with the risk of AIDS at diagnosis ([AIDSAD], defined as a CD4 count < 200 cells/µL) in patients with HIV subtype B, CRF01_AE, and CRF07_BC. Of the 20,663 sequences, 9,156 (44.3%) were CRF01_AE. CRF07_BC was responsible for 28.3% of infections, followed by B (13.9%). In multivariable analysis, the risk of AIDSAD differed significantly according to HIV subtype (OR for CRF07_BC vs. B: 0.46, 95% CI 0.39─0.53), age (OR for ≥ 65 years vs. < 18 years: 4.3 95% CI 1.81─11.8), and transmission risk groups (OR for men who have sex with men vs. heterosexuals: 0.67 95% CI 0.6─0.75). These findings suggest that HIV diversity in China is constantly evolving and gaining in complexity. CRF07_BC is less pathogenic than subtype B, while CRF01_AE is as pathogenic as B.

## Introduction

China has a slowly increasing HIV epidemic, with 64,170, 71,204, and 63,154 new cases in 2018, 2019, and 2020, respectively, and 818,360 individuals living with HIV at the end of 2020^1^. During the first two decades of the epidemic (1985–2005), most HIV cases were concentrated in the injection drug users (IDU [44.2%]) and former blood donors (29.6%), but since 2006, there has been a clear expansion in the number of the HIV cases in heterosexuals and men who have sex with men (MSM). In 2019, heterosexuals and MSM accounted for 73.8% and 23.3% of new diagnoses, respectively, with IDU accounting for 3.4%^2^. Understanding the increase in HIV diversity within China is not only of epidemiological interest but also has far-reaching clinical implications^3–9^.

One of the fascinating findings concerning the HIV subtype in China is the belief that CRF01_AE progresses faster than CRF07_BC^10–14^. However, these studies were limited by small sample sizes and failed to adjust for important confounding factors. Worldwide, findings consistently indicate that the rates of disease progression among different HIV subtypes are, in descending order, subtype C>D>CRF01_AE>G>A^15–24^. Although subtype B is the most studied because of its predominance in North America and Europe, it is absent in this comparison chain.

When comparing subtype B with non-B strains using non-B as the comparator, it is assumed that all subtypes except for B progress equally, which is obviously not the case. To date, no previous studies have been sufficiently large to directly compare subtype B with other single subtypes^15–24^. The latest national HIV epidemiology study in China was conducted in 2006 and was published in 2012^4^. Fourteen years have passed, and the China’s epidemic has changed. In this study, HIV disease progression was compared between patients infected with subtype B, CRF01_AE, and CRF07_BC in the largest HIV molecular epidemiology study ever conducted in China.

## Results

### Study population

**Figure 1:**
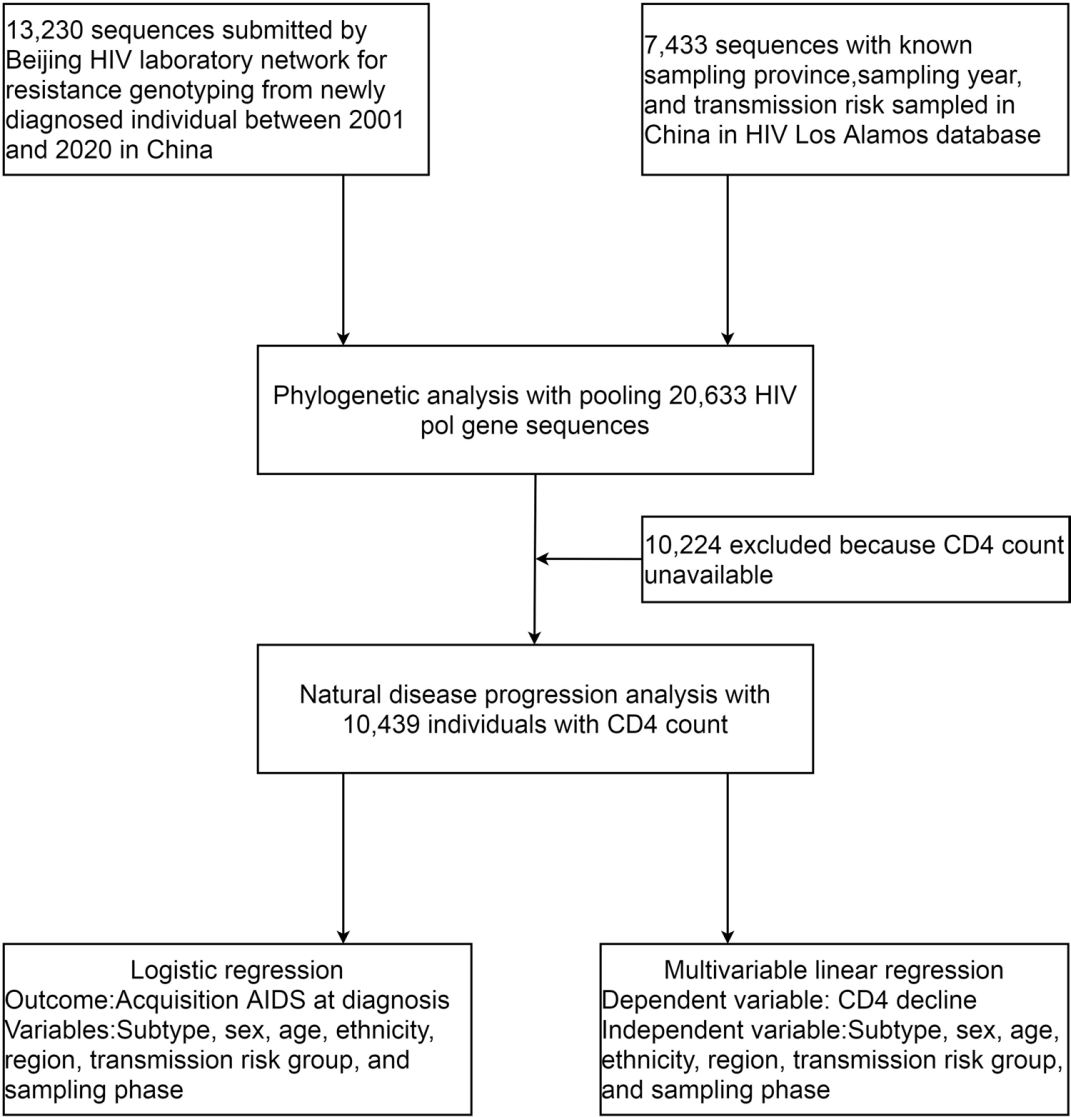
Study profile.

HIV *pol* sequences generated from 13,230 patient specimens submitted by the Beijing HIV laboratory network (BHLN) for HIV transmitted drug resistance (TDR) genotyping between 2001 and 2020 were analyzed. A total of 7433 *pol* sequences sampled in China were retrieved, of which the province of origin, transmission risk group and sampling year were available from the Los Alamos HIV sequence database (LANL). In all, 20,663 aligned HIV *pol* sequences were used in this analysis, each representing a distinct HIV-positive individual (Fig. 1). These data were collected between 1994 and 2020 from 31 provinces of China. Most participants were men (94.2%) of Han ethnicity (93.1%). The median age was 32 years (interquartile range [IQR] 26–42). Where available, the overall median baseline CD4 count was 338 cells/µL (IQR 208–475). The transmission risk group was predominantly MSM (66.6%), followed by heterosexual (23.3%) and IDU (7.7%) (Table 1).

**Table 1:** Baseline characteristic by sampling phase.

	1994–2005	2006–2008	2009–2011	2012–2014	2015–2017	2018–2020	Total	P value
**Sex**								
Men	169(70.71)	1162(86.07)	4162(95.68)	4714(96.5)	3651(95.48)	3293(92.5)	17,151(94.19)	< 0.05
Women	70(29.29)	188(13.93)	188(4.32)	171(3.5)	173(4.52)	267(7.5)	1057(5.81)	
Age (years)^a^	32(26–38)	31(25–38)	31(26–39)	31(26–40)	31(26–44)	33(27–45)	32(26–42)	NS
**Ethnicity**								
Han	173(77.58)	756(76.06)	1790(91.42)	2505(93.54)	3648(95.57)	3442(96.69)	12,314(93.08)	< 0.05
Uyghur	40(17.94)	139(13.98)	54(2.76)	29(1.08)	27(0.71)	6(0.17)	295(2.23)	
Yi	2(0.9)	35(3.52)	38(1.94)	34(1.27)	20(0.52)	12(0.34)	141(1.07)	
Other	8(3.59)	64(6.44)	76(3.88)	110(4.11)	122(3.2)	100(2.81)	480(3.63)	
CD4 count(cells per µl)^b^	299(181–402)	330(209–460)	345(202–455)	365(235–510)	326(204–462)	323(200–470)	338(208–475)	NS
**Transmission risk groups**								
Heterosexual	149(29.22)	616(31.4)	1045(19.27)	911(17.34)	837(21.96)	1227(34.51)	4785(23.32)	< 0.05
MSM	50(9.8)	742(37.82)	3581(66.03)	4120(78.42)	2867(75.23)	2295(64.54)	13,655(66.56)	
Injecting drug user	111(21.76)	418(21.3)	712(13.13)	203(3.86)	107(2.81)	34(0.96)	1585(7.73)	
Blood transfusion	190(37.25)	176(8.97)	79(1.46)	16(0.3)	0(0)	0(0)	461(2.25)	
Mother to Child	10(1.96)	10(0.51)	6(0.11)	4(0.08)	0(0)	0(0)	30(0.15)	
Region	y199405	y200608	y200911	y201214	y201517	y201820		< 0.05
North	104(19.85)	554(27.62)	1250(22.97)	1721(32.73)	1942(50.78)	1719(48.41)	7290(35.38)	
North-east	78(14.89)	104(5.18)	513(9.43)	393(7.47)	525(13.73)	561(15.8)	2174(10.55)	
East	79(15.08)	295(14.71)	1155(21.22)	1386(26.36)	461(12.06)	437(12.31)	3813(18.51)	
Central-south	148(28.24)	707(35.24)	1196(21.98)	1086(20.65)	464(12.13)	438(12.33)	4039(19.6)	
South-west	71(13.55)	162(8.08)	1180(21.68)	517(9.83)	234(6.12)	216(6.08)	2380(11.55)	
North-west	44(8.4)	184(9.17)	148(2.72)	155(2.95)	198(5.18)	180(5.07)	909(4.41)	
**Subtype**								
B	250(47.71)	602(29.86)	843(15.39)	570(10.84)	377(9.86)	235(6.6)	2877(13.92)	< 0.05
C	22(4.2)	31(1.54)	105(1.92)	55(1.05)	27(0.71)	19(0.53)	259(1.25)	
01_AE	103(19.66)	679(33.68)	2423(44.23)	2586(49.16)	1797(46.98)	1568(44.04)	9156(44.31)	
07_BC	89(16.98)	486(24.11)	1639(29.92)	1424(27.07)	1108(28.97)	1096(30.79)	5842(28.27)	
08_BC	26(4.96)	103(5.11)	70(1.28)	93(1.77)	33(0.86)	32(0.9)	357(1.73)	
55_01B	1(0.19)	28(1.39)	108(1.97)	149(2.83)	66(1.73)	99(2.78)	451(2.18)	
Minor	14(2.67)	38(1.88)	115(2.1)	94(1.79)	101(2.64)	137(3.85)	499(2.41)	
URF	19(3.63)	49(2.43)	175(3.19)	289(5.49)	316(8.26)	374(10.51)	1222(5.91)	

Data are n (%), or median (IQR).

MSM, men who have sex with men; URF, unique recombinant form.

North, Beijing, Tianjin, Hebei, Shanxi, and Inner Mongolia. North-east, Liaoning, Jilin, and Heilongjiang. East, Shanghai, Jiangsu, Zhejiang, Anhui, Fujian, Jiangxi, and Shandong. Central- south, Henan, Hubei, Hunan, Guangdong, Guangxi, and Hainan. South-west, Chongqing, Sichuan, Guizhou, Yunnan, and Tibet. North-west, Shann'xi, Gansu, Qinghai, Ningxia, and Sinkiang.

Minor, A1, D, F1, G, H, CRF02_AG, CRF03_AB, CRF06_cpx, CRF15_01B, CRF18_cpx, CRF24_BG, CRF33_01B, CRF55_01B, CRF57_BC, CRF58_01B, CRF59_01B, CRF61_BC, CRF62_BC, CRF63_02A1, CRF64_BC, CRF65_cpx, CRF67_01B, CRF68_01B, CRF78_cpx, CRF79_0107, CRF82_cpx, CRF83_cpx, CRF85_BC, CRF86_BC, CRF87_cpx, CRF88_BC, and CRF96_cpx.

^a^13,216.

^b^10,439.

### HIV subtype distribution

A total of 38 HIV subtypes and CRF were identified in the study. CRF01_AE, CRF07_BC, subtype B, URF, CRF55_01B, CRF08_BC, and subtype C (seven common subtypes) were the seven predominant HIV subtypes circulating in China, accounting for 44.3%, 28.3%, 13.9%, 5.9%, 2.2%, 1.7%, and 1.3% of all infections, respectively (Table 2).

**Table 2:** Subtype assignment by selected characteristics.

Characteristic	B		C		01_AE		07_BC		08_BC		55_01B		Minor		URF		Total	
	n	%	n	%	n	%	n	%	n	%	n	%	n	%	n	%	n	%
**Sex**																		
Men	2217	12.93	79	0.46	8201	47.82	4762	27.77	104	0.61	425	2.48	361	2.1	1002	5.84	17,151	100
Women	266	25.17	33	3.12	284	26.87	323	30.56	55	5.2	8	0.76	53	5.01	35	3.31	1057	100
**Age at diagnosis (years)**																		
< 18	33	36.67	3	3.33	20	22.22	26	28.89	1	1.11	1	1.11	3	3.33	3	3.33	90	100
18–24	222	10.63	10	0.48	987	47.27	661	31.66	12	0.57	31	1.48	40	1.92	125	5.99	2088	100
25–44	1246	15.02	50	0.6	3866	46.59	2225	26.81	87	1.05	117	1.41	167	2.01	540	6.51	8298	100
45–64	391	16.24	31	1.29	971	40.32	635	26.37	36	1.5	50	2.08	107	4.44	187	7.77	2408	100
≥ 65	44	13.25	5	1.51	111	33.43	114	34.34	11	3.31	5	1.51	13	3.92	29	8.73	332	100
**Ethnicity**																		
Han	1854	15.06	89	0.72	5734	46.56	3135	25.46	130	1.06	196	1.59	320	2.6	856	6.95	12,314	100
Uyghur	8	2.71	1	0.34	5	1.69	277	93.9	0	0	0	0	2	0.68	2	0.68	295	100
Yi	3	2.13	2	1.42	5	3.55	121	85.82	4	2.84	0	0	0	0	6	4.26	141	100
Others	73	15.21	7	1.46	218	45.42	132	27.5	13	2.71	8	1.67	8	1.67	21	4.38	480	100
**Transmission risk groups**																		
Heterosexual	735	15.36	133	2.78	2041	42.65	1088	22.74	207	4.33	76	1.59	190	3.97	315	6.58	4785	100
MSM	1640	12.01	39	0.29	6856	50.21	3672	26.89	39	0.29	372	2.72	254	1.86	783	5.73	13,655	100
Injecting drug user	63	3.97	80	5.05	161	10.16	1018	64.23	105	6.62	3	0.19	36	2.27	119	7.51	1585	100
Blood transfusion	378	82	0	0	41	8.89	31	6.72	3	0.65	0	0	7	1.52	1	0.22	461	100
Mother to child	15	50	1	3.33	6	20	3	10	2	6.67	0	0	3	10	0	0	30	100
**Region**																		
North	3460	47.46	1738	23.84	49	0.67	101	1.39	1198	16.43	57	0.78	201	2.76	486	6.67	7290	100
North-east	1211	55.7	460	21.16	15	0.69	21	0.97	281	12.93	13	0.6	52	2.39	121	5.57	2174	100
East	2108	55.28	887	23.26	52	1.36	66	1.73	449	11.78	17	0.45	88	2.31	146	3.83	3813	100
Central-south	1592	39.42	1153	28.55	97	2.4	243	6.02	686	16.98	19	0.47	52	1.29	197	4.88	4039	100
South-west	480	20.17	1124	47.23	137	5.76	16	0.67	151	6.34	149	6.26	91	3.82	232	9.75	2380	100
North-west	279	30.69	472	51.93	6	0.66	4	0.44	94	10.34	2	0.22	13	1.43	39	4.29	909	100
**Period**																		
1994–2005	103	19.66	89	16.98	26	4.96	1	0.19	250	47.71	22	4.2	14	2.67	19	3.63	524	100
2006–2008	679	33.68	486	24.11	103	5.11	28	1.39	602	29.86	31	1.54	38	1.88	49	2.43	2016	100
2009–2011	2423	44.23	1639	29.92	70	1.28	108	1.97	843	15.39	105	1.92	115	2.1	175	3.19	5478	100
2012–2014	2586	49.16	1424	27.07	93	1.77	149	2.83	570	10.84	55	1.05	94	1.79	289	5.49	5260	100
2015–2017	1797	46.98	1108	28.97	33	0.86	66	1.73	377	9.86	27	0.71	101	2.64	316	8.26	3825	100
2018–2020	1568	44.04	1096	30.79	32	0.9	99	2.78	235	6.6	19	0.53	137	3.85	374	10.51	3560	100

MSM, men who have sex with men; URF, unique recombinant form.

North, Beijing, Tianjin, Hebei, Shanxi, and Inner Mongolia. North-east, Liaoning, Jilin, and Heilongjiang. East, Shanghai, Jiangsu, Zhejiang, Anhui, Fujian, Jiangxi, and Shandong. Central- south, Henan, Hubei, Hunan, Guangdong, Guangxi, and Hainan. South-west, Chongqing, Sichuan, Guizhou, Yunnan, and Tibet. North-west, Shann'xi, Gansu, Qinghai, Ningxia, and Sinkiang.

Minor, A1, D, F1, G, H, CRF02_AG, CRF03_AB, CRF06_cpx, CRF15_01B, CRF18_cpx, CRF24_BG, CRF33_01B, CRF55_01B, CRF57_BC, CRF58_01B, CRF59_01B, CRF61_BC, CRF62_BC, CRF63_02A1, CRF64_BC, CRF65_cpx, CRF67_01B, CRF68_01B, CRF78_cpx, CRF79_0107, CRF82_cpx, CRF83_cpx, CRF85_BC, CRF86_BC, CRF87_cpx, CRF88_BC, and CRF96_cpx.

Additional clades including subtypes A1, D, F1, G, H, CRF02_AG, CRF03_AB, CRF06_cpx, CRF15_01B, CRF18_cpx, CRF24_BG, CRF33_01B, CRF55_01B, CRF57_BC, CRF58_01B, CRF59_01B, CRF61_BC, CRF62_BC, CRF63_02A1, CRF64_BC, CRF65_cpx, CRF67_01B, CRF68_01B, CRF78_cpx, CRF79_0107, CRF82_cpx, CRF83_cpx, CRF85_BC, CRF86_BC, CRF87_cpx, CRF88_BC, and CRF96_cpx (minor subtypes) were present in less than 1.0% of individuals. Of the subtypes, the combined prevalence of CRF01_AE, CRF07_BC, and subtype B (three major subtypes) remained steady at just over 85%, while the foreign subtypes (subtypes that originated and circulated mainly in foreign countries, including subtypes A1, D, F1, G, H, CRF 02_AG, CRF03_AB, CRF06_cpx, CRF15_01B, CRF18_cpx, CRF24_BG, CRF33_01B, CRF58_01B, CRF63_02A1, CRF_82cpx, and CRF_83cpx) constituted only 3% of all the subtypes.

**Figure 2:**
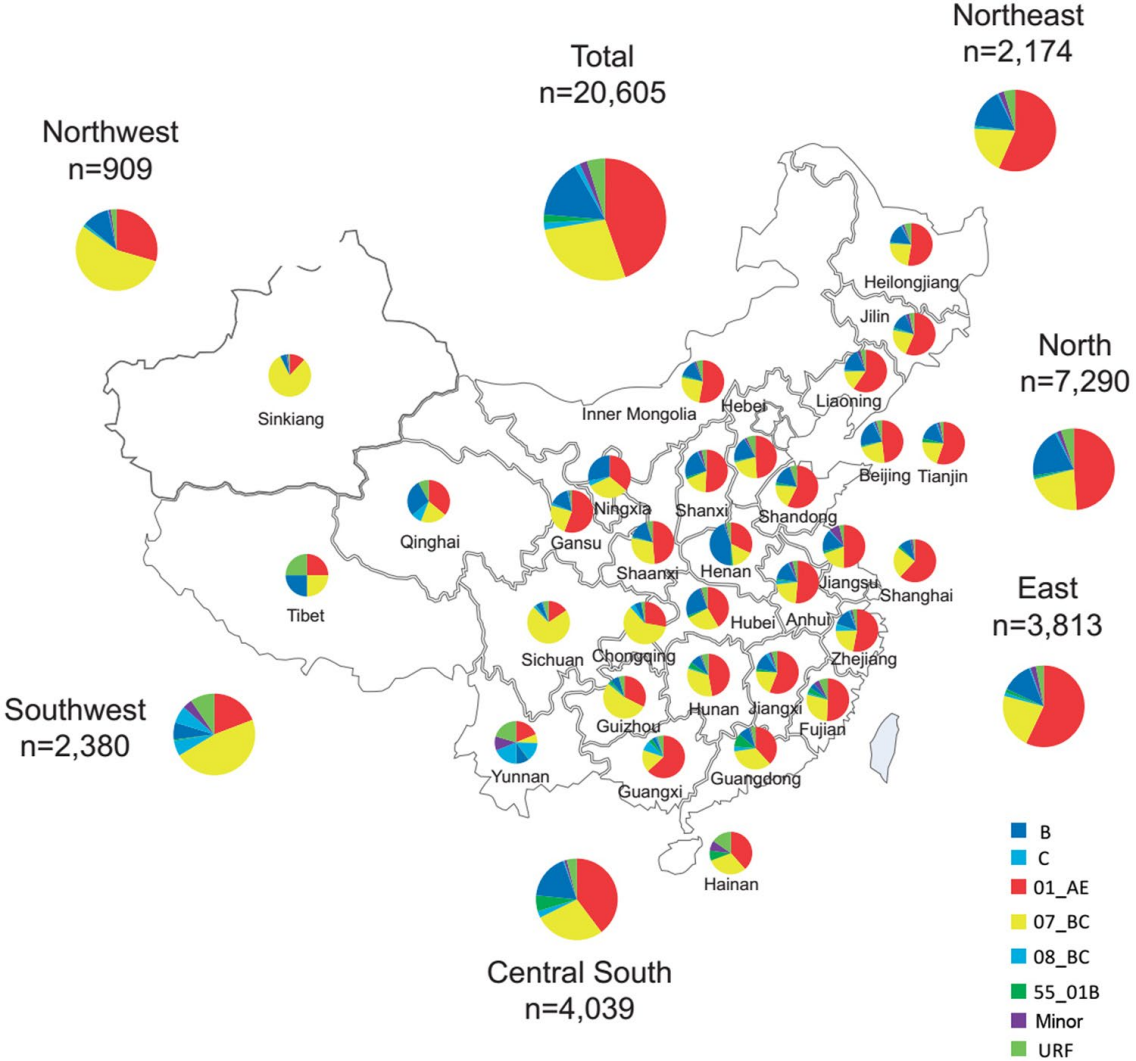
Geographical distribution of HIV subtype. Seven common subtypes, CRF01_AE, CRF07_BC, subtype B, URF, CRF55_01B, CRF08_BC, and subtype C. Samples were from 31 provinces of China. North, Beijing, Tianjin, Hebei, Shanxi, and Inner Mongolia; Northeast, Liaoning, Jilin, and Heilongjiang; East, Shanghai, Jiangsu, Zhejiang, Anhui, Fujian, Jiangxi, and Shandong; Central-south, Henan, Hubei, Hunan, Guangdong, Guangxi, and Hainan; Southwest, Chongqing, Sichuan, Guizhou, Yunnan, and Tibet; Northwest, Shann'xi, Gansu, Qinghai, Ningxia, and Sinkiang.

The prevalence of subtypes varied significantly according to sex, age, ethnicity, transmission risk group, and region. Table 2 shows the subtype diversity within the demographic subgroups. Women had a greater prevalence of infection with subtype B and a lower prevalence of infection with CRF01_AE and URF. Older individuals (≥ 65 years) tended to have lower prevalence of infection with CRF01_AE. Genotypic diversity was the greatest among heterosexuals, in which 35 HIV genotype categories were detected, the most prevalent being CRF01_AE. MSM included 21 subtypes, of which CRF01_AE was the most prevalent. IDU most likely had CRF07_BC. Individuals of Uyghur and Yi ethnicity predominantly, though not exclusively, were infected with the CRF07_BC virus. Figure 2. illustrates the regional distributions of the seven common subtypes HIV strains. CRF07_BC was more prevalent in the southwest and northwest regions. In the other four regions, the subtype with the highest prevalence was CRF01_AE. Of note, a significantly high prevalence of URF was detected in the southwest region (9.6%).The prevalence of comorbidites for individuals with HIV subtype B, CRF01_AE, and CRF07_BC were 14.0% (11.7–16.5%), 5.5% (2.6–6.4%), and 2.7% (1.9–3.5%), respectively.

### HIV subtype temporal trends

Table 1 presents the temporal trends for the seven common subtypes. The prevalence of CRF01_AE increased from 19.7% to 49.2% between the phase 1994–2005 and 2012–2014 and remained high. A similar trend was observed for CRF07_BC. Interestingly, the prevalence of subtype B decreased from 47.7% in phase 1994–2005 to 6.6% in 2018–2020. Time trends were also examined by sex, age, ethnicity, transmission risk group, and region (Supplementary Table S1–5).

### Phylogenetic analysis

Phylogenetic analysis revealed that the sequences from both sources were intermixed, suggesting that both sampling frames were drawn from the same overall population (Supplementary Figs. S1-3). Three, seven, and four distinct clusters were identified within subtype B, CRF01_AE, and CRF07_BC, respectively, which included 14,578 individuals (81.6% of all patients infected with the three major subtypes). The clusters have been named based on a previous numbering system^25^ and with the addition of new clusters in the current study. The cluster size ranged from 175–2964 individuals. Most clusters were MSM dominated (10 of 14). Supplementary Table S6 presents the detailed characteristics of these clusters.

### CRF07_BC progressed slower than subtype B

**Figure 3:**
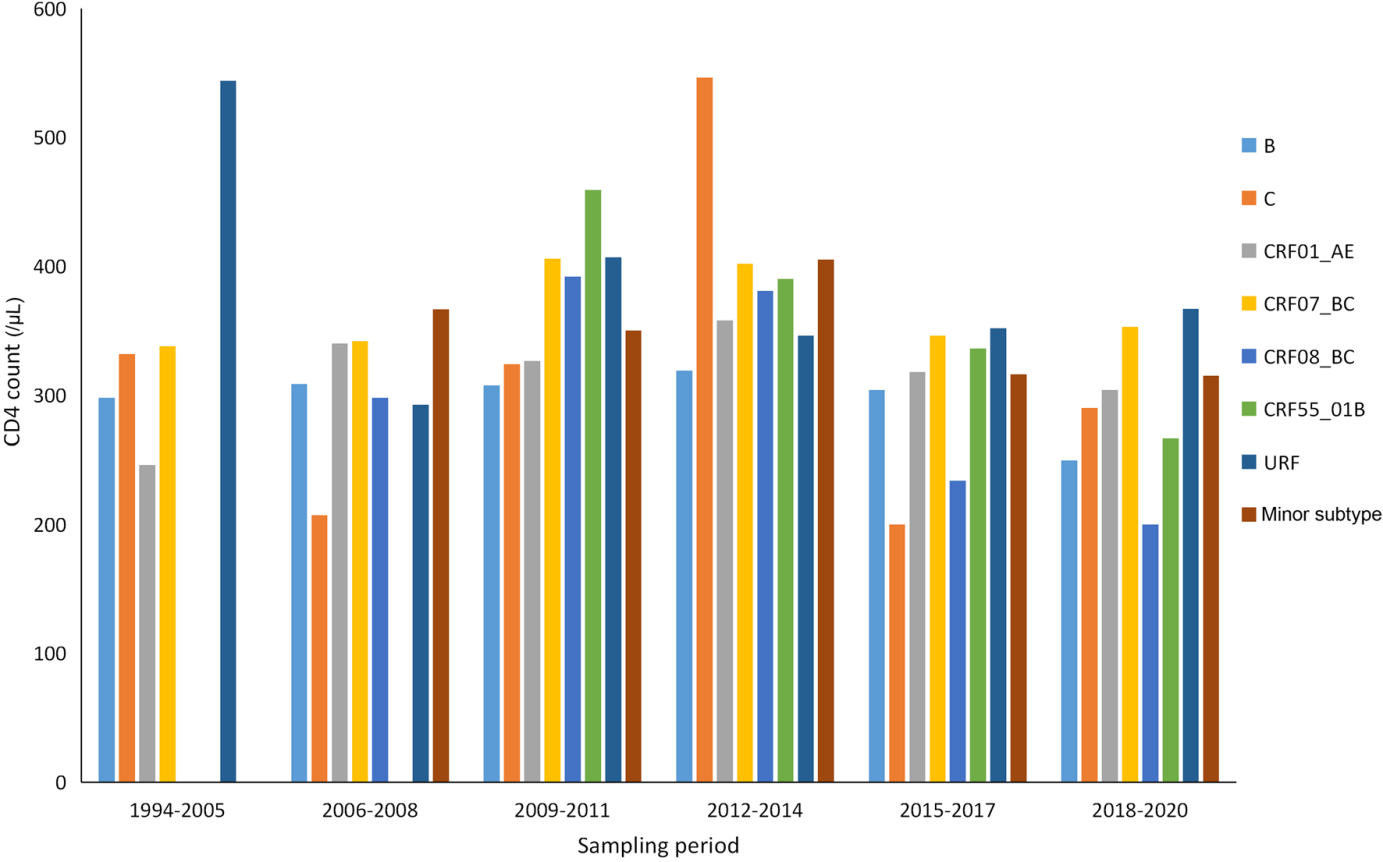
The CD4 count of the seven common subtypes.

Untreated HIV infections are characterized by a progressive decline in the number of CD4 cells, resulting in CD4 cell decline being recognized as one of the major markers of the rate of HIV disease progression. Most previous studies used the time from infection to the diagnosis of AIDS or the rate of CD4 loss per year to evaluate the rate of disease progression^15–24^. Both methods rely on an accurate determination of the date of HIV acquisition. As the date of HIV acquisition was not available for the majority of our study participants, we could not determine the rates of HIV disease progression with precision. However, the unavailability of the date of HIV acquisition did not prevent the direct comparison of disease progression between the three major subtypes on a population level. Though the time between HIV infection and diagnosis inevitably varies substantially among individuals, we believe that the interval was well-matched between the three major subtypes. In other words, they have identical median times. We aimed to compare the natural rate of CD4 decline between the three major subtypes within this interval. The interval between infection and diagnose is also part of nature disease history. Our analysis was based on the hypothesis that if the three major subtypes progress equally, there should be no difference in their median CD4 counts at the time of diagnosis on a population level. We defined origin time as the estimated date of HIV acquisition and the end time as the date of HIV diagnosis. We defined disease progression as the decline in CD4 count from the time of HIV acquisition to the time of HIV diagnosis. Therefore, our analysis was limited to the individuals with available CD4 counts. CD4 counts of the seven common subtypes were compared overall and by sampling phase. The CD4 count of CRF07_BC was always significantly higher than those of subtype B and CRF01_AE (*P* < 0.01) (Fig. 3). Therefore, we empirically hypothesized that disease progression in these subtypes could be different. Table 3 reports the association between patient characteristics and laboratory acquired immunodeficiency syndrome ([AIDS], defined as a CD4 count < 200 cells/µL) at diagnosis (AIDSAD).

**Table 3:** Risk factors for slower progression to AIDSAD in Chinese patients.

	Number of sequences	AIDSAD prevalence^a^	Univariable analysis^b^		Multivariable analysis^c^	
			OR(95% CI)	P value	OR(95% CI)	P value
**Sex**						
Men	9681	2210(22.83)	Reference			
Women	758	210(27.7)	1.29(1.1–1.53)	0.002	0.86(0.71–1.04)	0.12
**Age at diagnosis(years) group**						
< 18	48	10(20.83)	Reference			
18–24	1625	207(12.74)	0.55(0.12–1.19)	0.1	1.11(0.48–2.94)	0.82
25–44	6559	1463(22.31)	1.09(0.28–2.32)	0.81	2.05(0.9–5.4)	0.11
45–64	1962	648(33.03)	1.87(0.56–4)	0.08	3.36(1.47–8.87)	0.007
≥ 65	240	91(37.92)	2.32(1.14–5.13)	0.03	4.32(1.81–11.75)	0.002
**Ethnicity**						
Han	9879	2310(23.38)	Reference			
Uyghur	97	8(8.25)	0.29(0.13–0.57)	< 0.001	0.49(0.21–1.02)	0.08
Yi	90	7(7.78)	0.28(0.12–0.56)	0.001	0.43(0.17–0.9)	0.04
Other	373	95(25.47)	1.12(0.88–1.41)	0.35	1.18(0.92–1.5)	0.19
**Transmission risk group**						
Heterosexual	2855	841(29.46)	Reference			
MSM	7086	1454(20.52)	0.62(0.56–0.68)	< 0.001	0.67(0.6–0.75)	< 0.001
Injecting drug user	312	51(16.35)	0.47(0.34–0.63)	< 0.001	0.85(0.59–1.2)	0.36
Blood transfusion	112	51(45.54)	2(1.36–2.93)	< 0.001	1.86(1.24–2.79)	0.003
Mother to child	10	4(40)	1.6(0.41–5.6)	0.47	3.36(0.7–15.3)	0.12
**Subtype**						
B	1508	452(29.97)	Reference			
C	72	20(27.78)	0.9(0.51–1.5)	0.69	0.82(0.47–1.39)	0.48
01_AE	4819	1259(26.13)	0.83(0.73–0.94)	0.003	0.94(0.82–1.07)	0.35
07_BC	2815	421(14.96)	0.41(0.35–0.48)	< 0.001	0.46(0.39–0.53)	< 0.001
08_BC	95	29(30.53)	1.03(0.65–1.59)	0.91	0.83(0.51–1.3)	0.42
55_01B	169	41(24.26)	0.75(0.51–1.07)	0.12	0.78(0.53–1.13)	0.2
URF	708	142(20.06)	0.59(0.47–0.72)	< 0.001	0.62(0.5–0.78)	< 0.001
Minor	253	56(22.13)	0.66(0.48–0.91)	0.01	0.63(0.45–0.86)	0.005
**Region**						
North	5061	1191(23.53)	Reference			
North-east	1486	338(22.75)	0.96(0.83–1.1)	0.53		
East	1251	293(23.42)	0.99(0.86–1.15)	0.93		
Central-south	1324	313(23.64)	1(0.87–1.16)	0.93		
South-west	752	168(22.34)	0.93(0.78–1.12)	0.47		
North-west	542	110(20.3)	0.83(0.66–1.03)	0.09		
**Sampling phase**						
1994–2005	71	20(28.17)	Reference			
2006–2008	574	133(23.17)	0.77(0.45–1.36)	0.35		
2009–2011	1537	372(24.2)	0.81(0.49–1.41)	0.45		
2012–2014	2562	516(20.14)	0.64(0.39–1.11)	0.1		
2015–2017	3101	735(23.7)	0.79(0.48–1.37)	0.38		
2018–2020	2594	644(24.83)	0.84(0.51–1.45)	0.52		

AIDSAD, AIDS at diagnose; OR, odds ratio; MSM, men who have sex with men; URF, unique recombinant form;

North, Beijing, Tianjin, Hebei, Shanxi, and Inner Mongolia. North-east, Liaoning, Jilin, and Heilongjiang. East, Shanghai, Jiangsu, Zhejiang, Anhui, Fujian, Jiangxi, and Shandong. Central- south, Henan, Hubei, Hunan, Guangdong, Guangxi, and Hainan. South-west, Chongqing, Sichuan, Guizhou, Yunnan, and Tibet. North-west, Shann'xi, Gansu, Qinghai, Ningxia, and Sinkiang.

Minor, A1, D, F1, G, H, CRF02_AG, CRF03_AB, CRF06_cpx, CRF15_01B, CRF18_cpx, CRF24_BG, CRF33_01B, CRF55_01B, CRF57_BC, CRF58_01B, CRF59_01B, CRF61_BC, CRF62_BC, CRF63_02A1, CRF64_BC, CRF65_cpx, CRF67_01B, CRF68_01B, CRF78_cpx, CRF79_0107, CRF82_cpx, CRF83_cpx, CRF85_BC, CRF86_BC, CRF87_cpx, CRF88_BC, and CRF96_cpx.

^a^Data are n(%).

^b^Univariable logistic regression analysis.

^c^Multivariable logistic regression analysis.

In univariable logistic analyses, the risk of AIDSAD was significantly associated with sex, age, transmission risk group, and HIV subtype. After adjustment for these factors in the multivariable analysis, patients infected with CRF07_BC had only less than half of the risk of AIDSAD than those infected with subtype B (as odds ratios [OR] 0.46, 95% CI 0.39–0.53). Patients aged 45 years or older had a higher risk of AIDSAD than did younger patients (< 18 years, [OR for individuals aged 45–64 years vs. < 18 years: 3.36, 95% CI 1.47–8.87; OR for ≥ 65 vs. < 18 years:4.32, 95% CI 1.81–11.75]). The risk of AIDSAD was lower in MSM than it was for in heterosexual patients (OR 0.67, 95% CI 0.6–0.75). The Yi ethnicity was associated with a lower risk of AIDSAD (OR 0.43, 95% CI 0.17–0.9); however, the sample size was very small. Three sensitivity analyses, excluding heterosexuals, MSM, and IDU, were performed, and the outcomes were consistent with those obtained from the whole population (Supplementary Table S7–9). We also analyzed the decline in CD4 count between the time of HIV infection and diagnosis using multivariable linear regression (MLR). The results were consistent with those obtained by logistic analysis (Supplementary Tables S10–12).

## Discussion

To our knowledge, this is the largest study to date reporting on the national distribution and trends of HIV subtypes in China, with a sample size of over 20,000, and spanning 1994–2020^4^. These data showed that the HIV epidemic in China exhibited some of the greatest global genetic diversity, consisting of 38 HIV subtypes. The only other country to match China is the United States, which has approximately 15 subtypes^6,7^. This high and sharp increase in HIV subtype diversity in China is consistent with evidence from most regions of the world^3–9^.Although there were variations in the prevalence of the three major subtypes, the combined prevalence of these subtypes was stable throughout the study period, suggesting they might be an indicator of equally stable HIV transmission in China. The data revealed that the previously described subtype compartmentalization^4^ no longer existed in the transmission risk group or was of diminished impact in the geographic region, but persisted in people of the Uyghur and Yi ethnicity, in which it was as strong as it ever. Global travel and acquisition of infections abroad, population floating, and domestic transmission all likely contribute to increasing HIV viral diversity^4–9^.

The comparison of disease progression between subtype B and other subtypes has been hindered by the fact that there are few populations with multiple circulating subtypes, including subtype B^15–24^.The epidemic in China characterized by CRF01_AE, CRF07_BC, and subtype B co-circulating provides a unique opportunity for such a direct comparison. The data revealed that CRF07_BC progresses slower than subtype B, while CRF01_AE progresses as fast as subtype B. Consistent with the results of concerted action of seroconversion to AIDS and death in Europe (CASCADE)^16,17^, disease progression did not differ significantly by sex. The middle (45–64 years) and the older (≥ 65 years) age groups had the faster disease progression than the young (< 18 years). However, a lower disease progression was observed in MSM compare to that of heterosexuals. We hypothesize that this difference could be attributed to a shorter interval between seroconversion and diagnosis in MSM compared to that seen in heterosexuals, because most targeted HIV testing campaigns in China have always focused on the MSM population^2^.

The CRF07_BC strain is a relatively young HIV strain, that originated in IDU in China and is mainly confined to China^26^. During the past two decades, the number of individuals infected with CRF07_BC has undergone a significant increase in China, accounting for 38% of all infections in phase 2018–2020. Although it descends from the two most prevalent strains in the world (subtypes B and C), CRF07_BC displays many unique characteristics that differ from those of its parent strains. Li, et al. have also observed that individuals infected with CRF07_BC have a significantly higher baseline CD4 counts than those infected with CRF01_AE^14^. However, they did not realize that the higher CD4 counts could be regarded as a proxy of slower disease progression, nor did they generalize from their finding the conclusion that CRF07_BC progresses slower than CRF01_AE. We have previously shown that CRF07_BC has a lower TDR prevalence than subtype B and CRF01_AE (1.5% vs. 4.8% vs. 5.6%) respectively^27,28^. Ge et al.^29^ and Cao et al.^30^ have demonstrated that CRF07_BC is associated with better immune recovery in Chinese patients undergoing antiretroviral treatment (ART) compared to that of patients infected with CRF01_AE. Taken together, these results support the hypothesis that CRF07_BC is less pathogenic than subtype B.

Before 2014, people in China tended to accept the viewpoint that the Chinese people infected with HIV will have approximately ten years AIDS-free time before they enter the AIDS phase, as reported by the CASCADE study^16,17^. In 2014, Li, et al. showed that infection with CRF01_AE is associated with faster disease progression in Chinese patients infected through the sexual transmission risk group compared to that of patients infected with non-CRF01_AE (most were CRF07_BC and subtype B)^10^. The time interval between seroconversion and AIDS was only 4.8 years for CRF01_AE. The non-difference in disease progression for CRF01_AE and subtype B in these findings suggested that the time from seroconversion and AIDS for subtype B was far shorter than that previously believed. Two explanations are suggested. First, since ethnicity has been proven to be a major determinant of disease progression^18^, HIV subtype B may progress faster in Han individuals in China than in Western individuals. Second, as Wertheim, et al. have suggested, HIV subtype B is experiencing natural selection to become more virulent^31^.

The current study has significant implications for clinical practice and policy-making. First, since approximately 60% of patients (subtype B plus CRF01_AE accounted for 58.2%) with new infections in China will progress to AIDS within 4.8 years, these findings justify early treatment. Second, results of this study necessitate subtype-specific monitoring and treatment guidelines. Patients with CRF07_BC may have a better prognostic treatment outcome. Third, in evaluating AIDS disease burden, the prevalence of CRF07_BC should be taken into account.

This study has several limitations. First, although it is the largest study of this kind, this study represents only approximately two percent of all individuals living with HIV in China. Thus, these findings might not be fully representative. Second, viral loads (VL) information was not included in the study, which did not permit the evaluation of the association between VL and subtype. However, this is a goal of a future study. Third, the biological mechanisms underlying these observations were not elucidated. On this point, Huang et al.^12^ have shown that patients infected with CRF07_BC have significantly lower VL than those of patients infected with subtype B, which may be due to the deletion of seven amino acids that overlap with the apoptosis-linked gene 2-interacting protein (Alix) protein-binding domain of the p6^*gag*^. Fourth, the infection time for most of the participants was unavailable, so the rate of CD4 count decline per year could not be assessed. Indeed, as China implemented the World Health Organization (WHO)’s ‘treat-all’, ‘treat-early’, and ‘treatment as prevention’ policy in 2016^32,33^, approximately 90% of individuals with HIV were treated with ART within the first year after diagnosis, making an evaluation of the natural disease progression was not only impractical, but unethical. This study provides a novel method to directly compare the rate of natural disease progression between subtypes, that is, the duration between the infection and the diagnosis as follow-up time, and to treat the follow-up time as a matching variable in multivariable logistic analysis. Fifth, the MSM population was most likely over-represented in the study sample. However, the original data, from which stratified and weighted results may be easily calculated, has been provided.

In summary, these results highlight a China HIV epidemic characterized by a high prevalence of CRF01_AE, CRF07_BC, and subtype B infections, with an overall increasing subtype diversity over the past 26 years, providing a unique opportunity to directly compare disease progression among the three subtypes. Disease progression was slower with CRF07_BC infection than with that of subtype B infection. Moreover, for the first time, it was shown that infections with CRF01_AE progressed as fast as those with subtype B. Future studies focusing on the effect of subtype on the outcome of ART, which include more confounding variables, such as VL, will help improve clinical practice and policymaking.

## Methods

### Study population and design

The study population consisted of two separate populations of HIV-infected individuals. The first group comprised all patients with the HIV TDR genotype, performed between 2001 and 2020 at the BHLN. BHLN is a national collaboration engaged in surveillance of HIV TDR in China^27,28^. These methods have been previously described. Briefly, approximately 40% of the samples from all individuals newly diagnosed with HIV infection by BHLN between 2001 and 2020 were randomly selected^27,28^. The BHLN takes part in maintaining the national HIV epidemiology database, which tracks everyone who receives a diagnosis of HIV infection in China and records the baseline CD4 count of all individuals with newly diagnosed HIV infection. The baseline CD4 count was the value from their CD4 count closest to the date on which their HIV infection was confirmed by western blot within one year. Baseline demographic data on sex, age, ethnicity, Hukou province, and the transmission risk group were retrieved from this database.

The second group included publicly available sequences from the LANL^34^. All the *pol* sequences sampled in China with known sampling provinces, sampling years, and transmission risk groups available in the database were downloaded (data available as of December 1, 2019).

### Phylogenetic analysis

Sequences were aligned using the BioEdit tool and the alignment was manually corrected according to the encoded reading frame. Duplicate sequences were discarded. If several sequences from the same patient were available in the database, only the oldest was retained. Long branch sequences were re-confirmed for their genotype, and those that were miscatalogued were eliminated from the study. A maximum likelihood phylogenetic tree was reconstructed with the merged dataset using the GTR + CAT nucleotide substitution model in FastTree 2.1^35^.The HIV subtype was inferred by automated subtyping using context-based modeling for expeditious typing (COMET)^36^, followed by phylogenetic analysis. Each sequence was assigned to one of eight subtypes, one of 102 circulating recombinant forms (CRF), or “unassigned.” An “unassigned” sequence was deemed a possible unique recombinant forms (URF)^6^.

### Cohort of natural disease progression

The BHLN may also be used as a cohort to study natural disease progression of HIV in China. The starting point of the study was set as the onset of the infection and the outcome was AIDSAD. The follow-up time was the duration between the starting point and the outcome. As the seroconversion time for most of the participants was unavailable, the follow-up time was unmeasurable. To solve this problem, the follow-up time was treated as a matching variable in cohort analysis, as we hypothesized that the distribution of the follow-up time was well matched within the same transmission risk group and roughly matched the study population as a whole. Three sensitivity analysis were performed by excluding heterosexuals, MSM, and IDU for the comparison of subtypes.

### Statistical analysis

For geographic location, participants were grouped into 31 provinces according to the Hukou. Hukou is a basic household registration system in China; this system officially identifies a person as a resident of an area and includes identifying information such as name, parents, spouse, and date of birth. These provinces were further divided into six regions according to their proximity and socio-economic status, in line with guidelines from the National Bureau of Statistics of China: north, northeast, east, central-south, southwest, and northwest. Six sampling phases were established:1994–2005, 2006–2008, 2009–2011, 2012–2014, 2015–2017, and 2018–2020. The earliest (1994–2005) phase encompassed more years to account for the relatively fewer data available in these years. The prevalence of subtype by sex, age, ethnicity, transmission risk group, Hukou province, and region was calculated and the subtype distribution trends over the six sampling phases were examined. Categorical data were compared using the chi-squared test and continuous data were compared using one-way analysis of variance, wherever appropriate.

Potential risk factors for acquiring AIDSAD were analyzed using logistic regression. Biologically plausible interactions were assessed in the multivariable model. Variables included sex, age (< 18, 18–24, 25–44, 45–64, and ≥ 65 years), ethnicity, region, subtype, transmission risk group, and sampling phase. In the model, a binary response was included, indicating the acquisition AIDSAD from each patient as an outcome. All variables were analyzed separately and the associated variables (*P* < 0.1) with their outcomes were entered into the multivariable model. The logistic results are expressed as OR with 95% confidence intervals (CI) and two-sided *P* values, where *P* < 0.05 was considered significant.

The decline in CD4 count between the time of HIV infection and diagnosis were analyzed using MLR. In the regression, the dependent variable was the difference in CD4 count between HIV infection and diagnosis, and the independent variables were all the variables selected in the logistic regression. The MLR results are presented as coefficients and *P* value. Since pre-infection CD4 counts were not measured, the reference median CD4 count in Chinese healthy adults was used^37^. All analyses were performed using R software (version 4.1.1; R Foundation, Vienna, Austria) and a listwise deletion was used to handle the missing data.

### Ethical issues

All analyses were performed on de-identified datasets to protect participants’ anonymity. The research ethics committee at the Beijing Center for Disease Prevention and Control approved this study, and all the methods in this study were performed in accordance with the approved guidelines. By law, consent was not required as these data were collected and analyzed in the course of routine public health surveillance.

## Supplementary Information

Supplementary Information.

## Data Availability

The datasets used and analyzed during the current study are available from the corresponding author on reasonable request.
