# Supplementary material for: CRF07_BC is associated with slow HIV disease progression in Chinese patients
Source: Sci Rep. 2022 Mar 8;12:3773. doi: 10.1038/s41598-022-07518-4 (PMC8904811; doi:10.1038/s41598-022-07518-4)
Supplement: Supplementary file 1 — Supplementary Information. [file 41598_2022_7518_MOESM1_ESM.pdf]

Supplementary Information

CRF07_BC is associated with slow HIV disease progression in Chinese patients

Jingrong Ye1,Jing Chen1,Juan Wang1,Yuncong Wang1, Hui Xing2¶*,Fengting Yu3, Lifeng Liu4, Yang Han5, Huihuang Huang6, Yi Feng2, Yuhua Ruan2, Minna Zheng7, Xinli Lu8, Xiaoli Guo9, Hong Yang10, Qi Guo11, Yi Lin12, Jianjun Wu13, Shouli Wu14, Yilong Tang15, Xiaoguang Sun16, Xiaobai Zou17, Guolong Yu18, Jianjun Li19, Quanhua Zhou20, Ling Su21, Lincai Zhang22, Zhan Gao23, Ruolei Xin1, Shufang He1, Conghui Xu1, Mingqiang Hao1, Yinxiao Hao1, Xianlong Ren1, Jie Li1, Lishi Bai1,Tianjun Jiang6, Tong Zhang4,Yiming Shao2¶*, Hongyan Lu1¶*

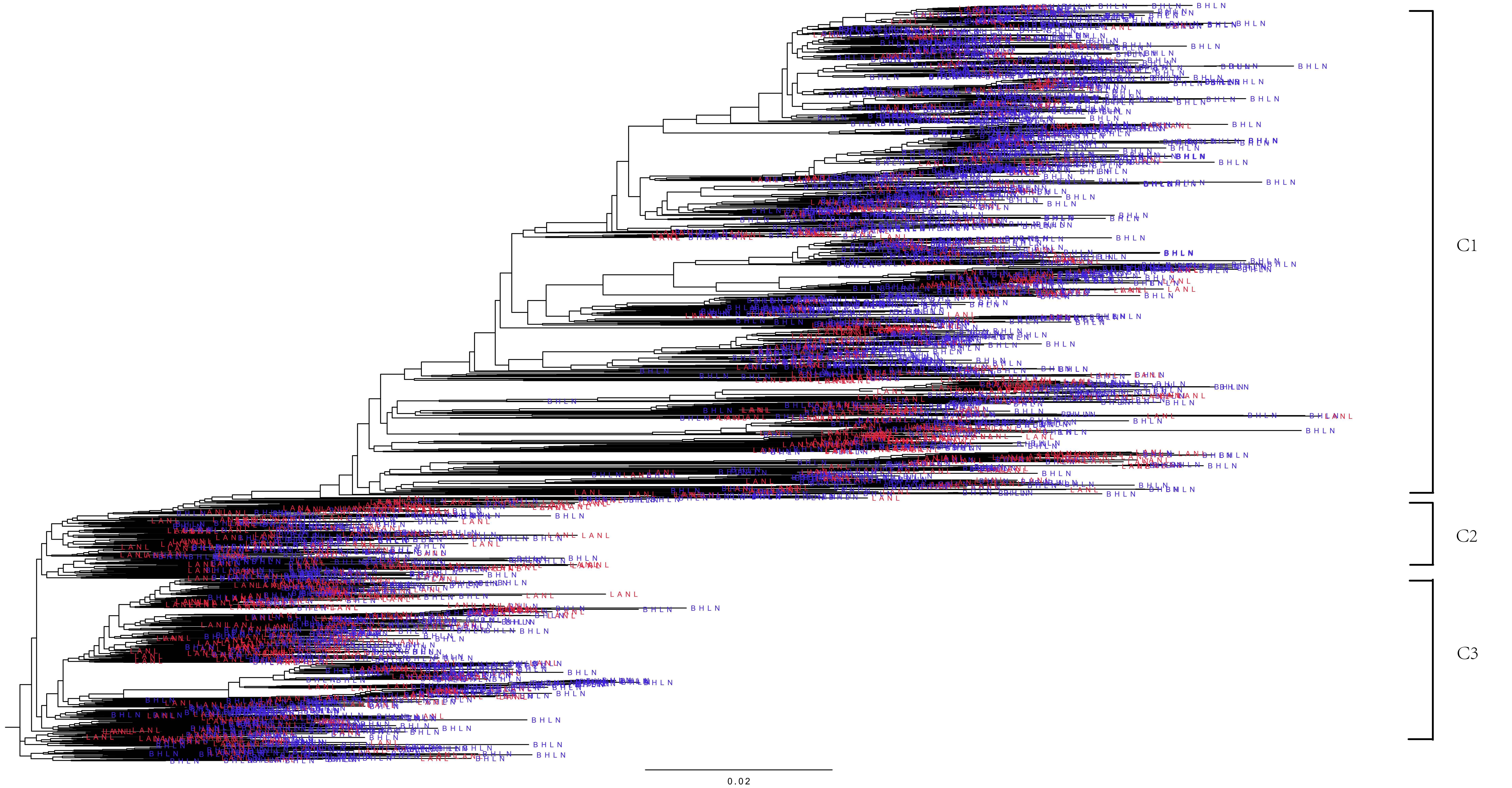
C1
C2
C3
0.02

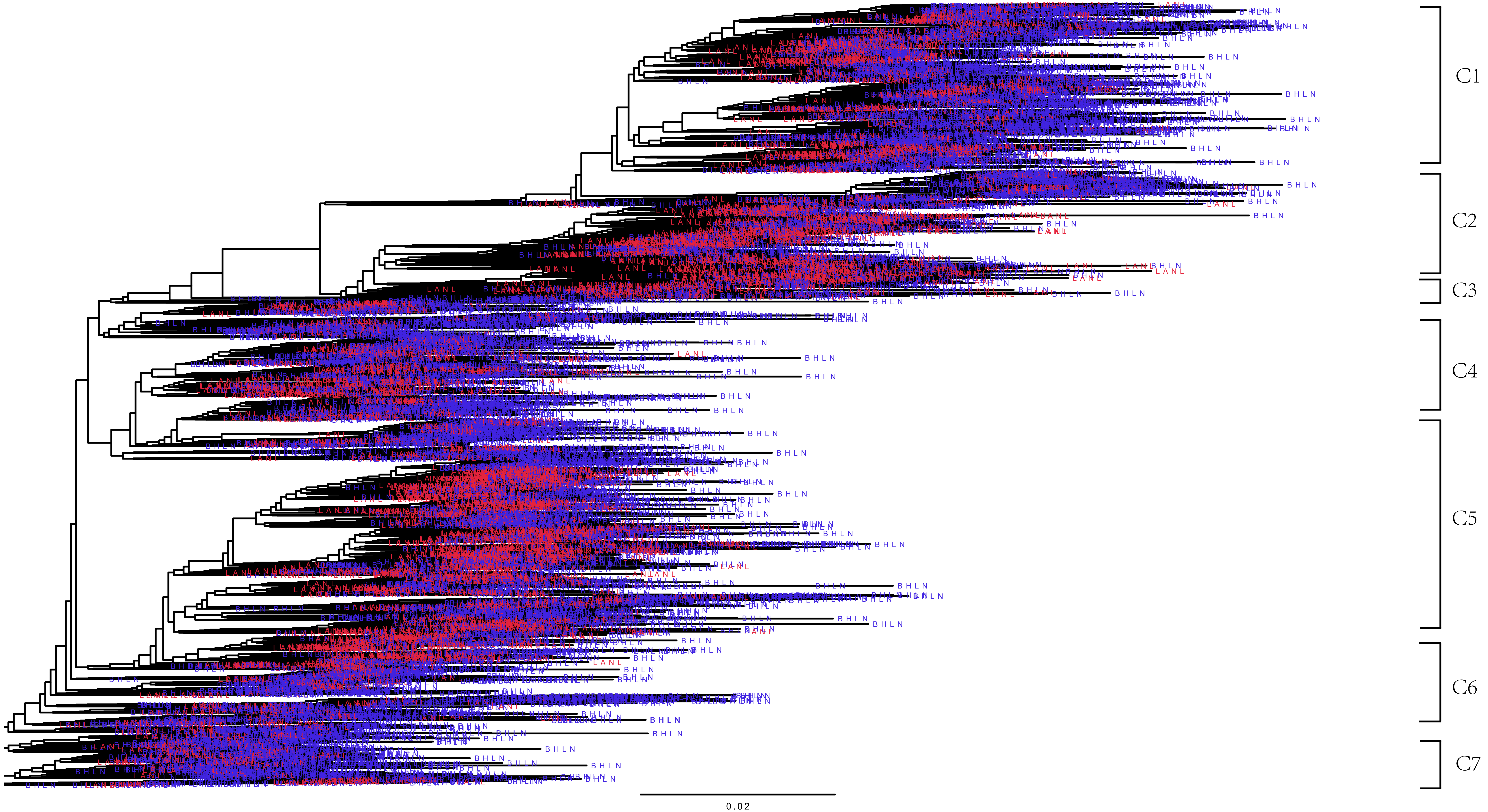
C1
C2
C3
C4
C5
C6
C7
B H L N
L A N L
0.02

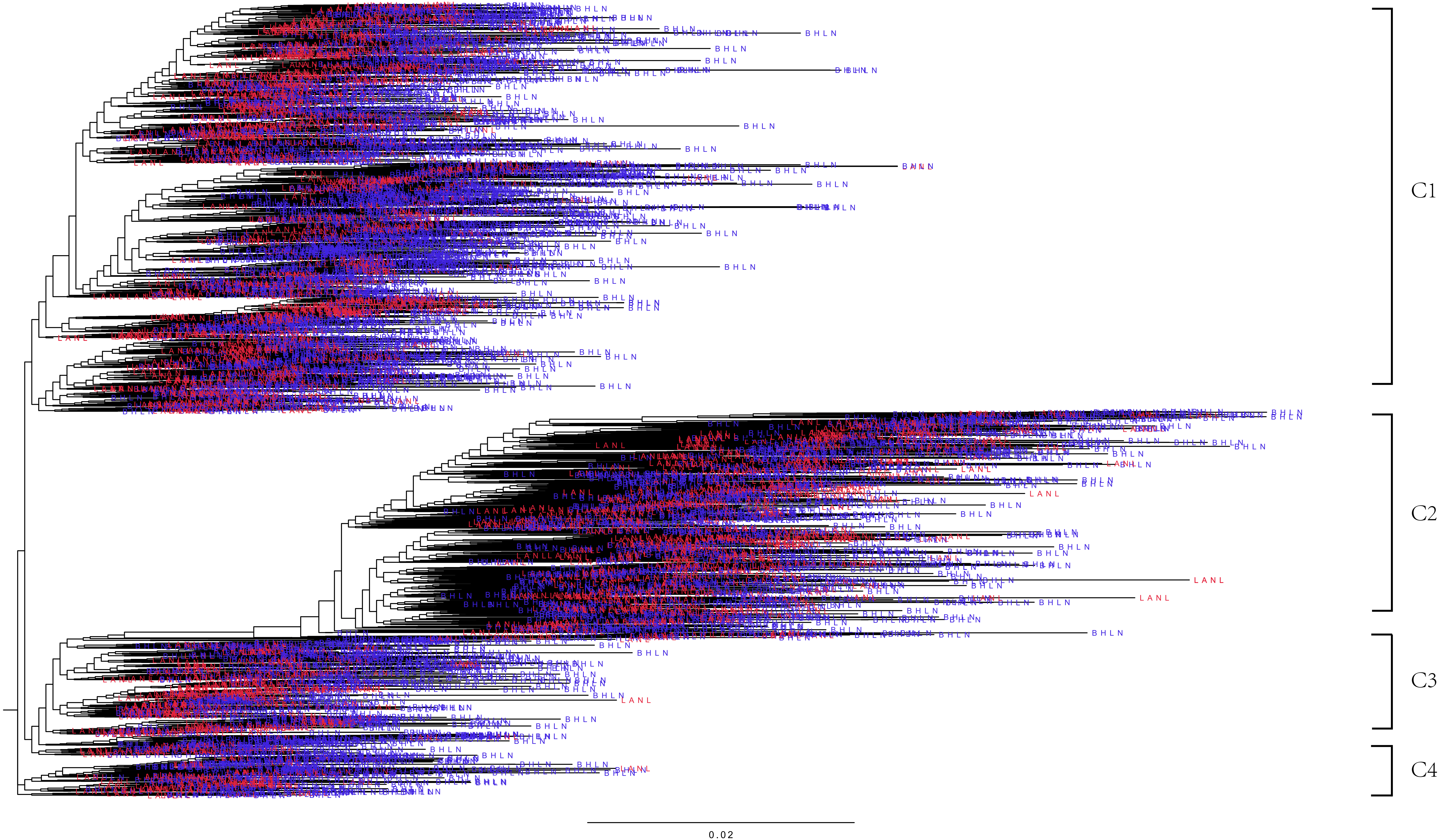
C1
C2
C3
C4
0.02
B H L N
L A N L

FigS1. The maximum likelihood phylogenetic tree of HIV subtype B *pol* sequences

The trees were constructed from nucleotide sequences corresponding to HXB2 nucleotide 2273 to 3276 using the GTR+CAT nucleotide substitution model in FastTree package. BHLN in blue indicate that the sequence is from Beijing HIV laboratory network(BHLN). LANL in red indicate that the sequence is Los Alamos HIV sequence database (LANL). A distance scale is shown at the bottom of the tree.

FigS2. The maximum likelihood phylogenetic tree of HIV CRF01_AE *pol* sequences

The trees were constructed from nucleotide sequences corresponding to HXB2 nucleotide 2273 to 3276 using the GTR+CAT nucleotide substitution model in FastTree package. BHLN in blue indicate that the sequence is from Beijing HIV laboratory network(BHLN). LANL in red indicate that the sequence is Los Alamos HIV sequence database (LANL). A distance scale is shown at the bottom of the tree.

FigS3. The maximum likelihood phylogenetic tree of HIV CRF07_BC *pol* sequences

The trees were constructed from nucleotide sequences corresponding to HXB2 nucleotide 2273 to 3276 using the GTR+CAT nucleotide substitution model in FastTree package. BHLN in blue indicate that the sequence is from Beijing HIV laboratory network(BHLN). LANL in red indicate that the sequence is Los Alamos HIV sequence database (LANL). A distance scale is shown at the bottom of the tree.

Table S1. HIV subtype trend for transmission risk group

	1994-2005	2006-2008	2009-2011	2012-2014	2015-2017	2018-2020	Total
Heterosexual							
01_AE	54(36.24)	259(42.05)	428(40.96)	420(46.1)	360(43.01)	520(42.38)	2041(42.65)
07_BC	17(11.41)	105(17.05)	187(17.89)	192(21.08)	234(27.96)	353(28.77)	1088(22.74)
08_BC	10(6.71)	54(8.77)	55(5.26)	48(5.27)	18(2.15)	22(1.79)	207(4.33)
55_01B	1(0.67)	1(0.16)	14(1.34)	9(0.99)	10(1.19)	41(3.34)	76(1.59)
B	45(30.2)	152(24.68)	196(18.76)	131(14.38)	109(13.02)	102(8.31)	735(15.36)
C	9(6.04)	12(1.95)	59(5.65)	27(2.96)	14(1.67)	12(0.98)	133(2.78)
Minor	6(4.03)	18(2.92)	43(4.11)	30(3.29)	31(3.7)	62(5.05)	190(3.97)
URF	7(4.7)	15(2.44)	63(6.03)	54(5.93)	61(7.29)	115(9.37)	315(6.58)
Total	149(100)	616(100.02)	1045(100)	911(100)	837(99.99)	1227(99.99)	4785(100)
MSM							
01_AE	14(28)	349(47.04)	1926(53.78)	2129(51.67)	1405(49.01)	1033(45.01)	6856(50.21)
07_BC	2(4)	108(14.56)	880(24.57)	1137(27.6)	815(28.43)	730(31.81)	3672(26.89)
08_BC	1(2)	2(0.27)	5(0.14)	11(0.27)	11(0.38)	9(0.39)	39(0.29)
55_01B	0(0)	27(3.64)	94(2.62)	139(3.37)	56(1.95)	56(2.44)	372(2.72)
B	33(66)	242(32.61)	547(15.28)	426(10.34)	260(9.07)	132(5.75)	1640(12.01)
C	0(0)	2(0.27)	5(0.14)	14(0.34)	11(0.38)	7(0.31)	39(0.29)
Minor	0(0)	6(0.81)	54(1.51)	54(1.31)	65(2.27)	75(3.27)	254(1.86)
URF	0(0)	6(0.81)	70(1.95)	210(5.1)	244(8.51)	253(11.02)	783(5.73)
Total	50(100)	742(100.01)	3581(99.99)	4120(100)	2867(100)	2295(100)	13655(100)
IDU							
01_AE	13(11.71)	51(12.2)	26(3.65)	31(15.27)	28(26.17)	12(35.29)	161(10.16)
07_BC	53(47.75)	260(62.2)	553(77.67)	84(41.38)	56(52.34)	12(35.29)	1018(64.23)
08_BC	13(11.71)	45(10.77)	9(1.26)	33(16.26)	4(3.74)	1(2.94)	105(6.62)
55_01B	0(0)	0(0)	0(0)	1(0.49)	0(0)	2(5.88)	3(0.19)
B	9(8.11)	13(3.11)	27(3.79)	6(2.96)	7(6.54)	1(2.94)	63(3.97)
C	12(10.81)	14(3.35)	41(5.76)	13(6.4)	0(0)	0(0)	80(5.05)
Minor	0(0)	8(1.91)	15(2.11)	10(4.93)	3(2.8)	0(0)	36(2.27)
URF	11(9.91)	27(6.46)	41(5.76)	25(12.32)	9(8.41)	6(17.65)	119(7.51)
Total	111(100)	418(100)	712(100)	203(100.01)	107(100)	34(99.99)	1585(100)
Blood transfusion							
01_AE	18(9.47)	7(3.98)	14(17.72)	2(12.5)	0(0)	0(0)	41(8.89)
07_BC	11(5.79)	8(4.55)	4(5.06)	8(50)	0(0)	0(0)	31(6.72)
08_BC	2(1.05)	0(0)	0(0)	1(6.25)	0(0)	0(0)	3(0.65)
55_01B	0(0)	0(0)	0(0)	0(0)	0(0)	0(0)	0(0)
B	154(81.05)	159(90.34)	60(75.95)	5(31.25)	0(0)	0(0)	378(82)
C	0(0)	0(0)	0(0)	0(0)	0(0)	0(0)	0(0)
Minor	5(2.63)	2(1.14)	0(0)	0(0)	0(0)	0(0)	7(1.52)
URF	0(0)	0(0)	1(1.27)	0(0)	0(0)	0(0)	1(0.22)
Total	190(99.99)	176(100.01)	79(100)	16(100)	0(0)	0(0)	461(100)
Mother to							

children							
01_AE	2(20)	1(10)	2(33.33)	1(25)	0(0)	0(0)	6(20)
07_BC	1(10)	0(0)	2(33.33)	0(0)	0(0)	0(0)	3(10)
08_BC	0(0)	2(20)	0(0)	0(0)	0(0)	0(0)	2(6.67)
55_01B	0(0)	0(0)	0(0)	0(0)	0(0)	0(0)	0(0)
B	6(60)	6(60)	1(16.67)	2(50)	0(0)	0(0)	15(50)
C	0(0)	0(0)	0(0)	1(25)	0(0)	0(0)	1(3.33)
Minor	1(10)	1(10)	1(16.67)	0(0)	0(0)	0(0)	3(10)
URF	0(0)	0(0)	0(0)	0(0)	0(0)	0(0)	0(0)
Total	10(100)	10(100)	6(100)	4(100)	0(0)	0(0)	30(100)

Data are n(%).

MSM, Man who have sex with man.

URF, unique recombinant form.

Minor,A1, D, F1, G, H, CRF02_AG, CRF03_AB, CRF06_cpx, CRF15_01B, CRF18_cpx, CRF24_BG, CRF33_01B, CRF55_01B, CRF57_BC, CRF58_01B, CRF59_01B, CRF61_BC, CRF62_BC, CRF63_02A1, CRF64_BC, CRF65_cpx, CRF67_01B, CRF68_01B, CRF78_cpx, CRF79_0107, CRF82_cpx, CRF83_cpx, CRF85_BC, CRF86_BC, CRF87_cpx, CRF88_BC, and CRF96_cpx.

Table S2. HIV subtype trend for region

	1994-2005	2006-2008	2009-2011	2012-2014	2015-2017	2018-2020	Total
North							
01_AE	15(14.42)	222(40.07)	608(48.64)	903(52.47)	950(48.92)	762(44.33)	3460(47.46)
07_BC	17(16.35)	71(12.82)	256(20.48)	397(23.07)	498(25.64)	499(29.03)	1738(23.84)
08_BC	3(2.88)	6(1.08)	5(0.4)	5(0.29)	12(0.62)	18(1.05)	49(0.67)
55_01B	0(0)	4(0.72)	7(0.56)	15(0.87)	30(1.54)	45(2.62)	101(1.39)
B	62(59.62)	221(39.89)	327(26.16)	261(15.17)	213(10.97)	114(6.63)	1198(16.43)
C	2(1.92)	10(1.81)	6(0.48)	13(0.76)	14(0.72)	12(0.7)	57(0.78)
Minor	4(3.85)	14(2.53)	18(1.44)	23(1.34)	58(2.99)	84(4.89)	201(2.76)
URF	1(0.96)	6(1.08)	23(1.84)	104(6.04)	167(8.6)	185(10.76)	486(6.67)
Total	104(100)	554(100)	1250(100)	1721(100.01)	1942(100)	1719(100.01)	7290(100)
North-east							
01_AE	17(21.79)	37(35.58)	351(68.42)	230(58.52)	275(52.38)	301(53.65)	1211(55.7)
07_BC	10(12.82)	11(10.58)	60(11.7)	82(20.87)	151(28.76)	146(26.02)	460(21.16)
08_BC	4(5.13)	7(6.73)	0(0)	0(0)	3(0.57)	1(0.18)	15(0.69)
55_01B	0(0)	1(0.96)	1(0.19)	3(0.76)	2(0.38)	14(2.5)	21(0.97)
B	36(46.15)	44(42.31)	78(15.2)	49(12.47)	43(8.19)	31(5.53)	281(12.93)
C	1(1.28)	2(1.92)	4(0.78)	2(0.51)	1(0.19)	3(0.53)	13(0.6)
Minor	7(8.97)	2(1.92)	11(2.14)	6(1.53)	11(2.1)	15(2.67)	52(2.39)
URF	3(3.85)	0(0)	8(1.56)	21(5.34)	39(7.43)	50(8.91)	121(5.57)
Total	78(99.99)	104(100)	513(99.99)	393(100)	525(100)	561(99.99)	2174(100.01)
East							
01_AE	30(37.97)	131(44.41)	713(61.73)	809(58.37)	234(50.76)	191(43.71)	2108(55.28)
07_BC	10(12.66)	41(13.9)	223(19.31)	360(25.97)	116(25.16)	137(31.35)	887(23.26)
08_BC	5(6.33)	23(7.8)	10(0.87)	7(0.51)	3(0.65)	4(0.92)	52(1.36)
55_01B	0(0)	0(0)	7(0.61)	29(2.09)	16(3.47)	14(3.2)	66(1.73)
B	27(34.18)	82(27.8)	159(13.77)	109(7.86)	38(8.24)	34(7.78)	449(11.78)
C	4(5.06)	3(1.02)	2(0.17)	2(0.14)	5(1.08)	1(0.23)	17(0.45)
Minor	1(1.27)	8(2.71)	24(2.08)	26(1.88)	15(3.25)	14(3.2)	88(2.31)
URF	2(2.53)	7(2.37)	17(1.47)	44(3.17)	34(7.38)	42(9.61)	146(3.83)
Total	79(100)	295(100.01)	1155(100.01)	1386(99.99)	461(99.99)	437(100)	3813(100)
Central-south							
01_AE	27(18.24)	236(33.38)	512(42.81)	460(42.36)	180(38.79)	177(40.41)	1592(39.42)
07_BC	4(2.7)	158(22.35)	333(27.84)	350(32.23)	162(34.91)	146(33.33)	1153(28.55)
08_BC	0(0)	49(6.93)	27(2.26)	15(1.38)	3(0.65)	3(0.68)	97(2.4)
55_01B	1(0.68)	23(3.25)	91(7.61)	97(8.93)	12(2.59)	19(4.34)	243(6.02)
B	112(75.68)	215(30.41)	175(14.63)	105(9.67)	50(10.78)	29(6.62)	686(16.98)
C	1(0.68)	5(0.71)	4(0.33)	4(0.37)	3(0.65)	2(0.46)	19(0.47)
Minor	2(1.35)	5(0.71)	15(1.25)	8(0.74)	11(2.37)	11(2.51)	52(1.29)
URF	1(0.68)	16(2.26)	39(3.26)	47(4.33)	43(9.27)	51(11.64)	197(4.88)
Total	148(100.01)	707(100)	1196(99.99)	1086(100.01)	464(100.01)	438(99.99)	4039(100.01)
South-west							

01_AE	13(18.31)	37(22.84)	169(14.32)	128(24.76)	69(29.49)	64(29.63)	480(20.17)
07_BC	8(11.27)	55(33.95)	687(58.22)	170(32.88)	103(44.02)	101(46.76)	1124(47.23)
08_BC	13(18.31)	17(10.49)	25(2.12)	64(12.38)	12(5.13)	6(2.78)	137(5.76)
55_01B	0(0)	0(0)	2(0.17)	3(0.58)	6(2.56)	5(2.31)	16(0.67)
B	11(15.49)	19(11.73)	78(6.61)	24(4.64)	12(5.13)	7(3.24)	151(6.34)
C	14(19.72)	8(4.94)	89(7.54)	33(6.38)	4(1.71)	1(0.46)	149(6.26)
Minor	0(0)	8(4.94)	43(3.64)	30(5.8)	3(1.28)	7(3.24)	91(3.82)
URF	12(16.9)	18(11.11)	87(7.37)	65(12.57)	25(10.68)	25(11.57)	232(9.75)
Total	71(100)	162(100)	1180(99.99)	517(99.99)	234(100)	216(99.99)	2380(100)
North-west							
01_AE	1(2.27)	16(8.7)	50(33.78)	56(36.13)	88(44.44)	68(37.78)	279(30.69)
07_BC	40(90.91)	149(80.98)	74(50)	65(41.94)	78(39.39)	66(36.67)	472(51.93)
08_BC	1(2.27)	1(0.54)	2(1.35)	2(1.29)	0(0)	0(0)	6(0.66)
55_01B	0(0)	0(0)	0(0)	2(1.29)	0(0)	2(1.11)	4(0.44)
B	2(4.55)	13(7.07)	18(12.16)	21(13.55)	21(10.61)	19(10.56)	94(10.34)
C	0(0)	2(1.09)	0(0)	0(0)	0(0)	0(0)	2(0.22)
Minor	0(0)	1(0.54)	3(2.03)	1(0.65)	3(1.52)	5(2.78)	13(1.43)
URF	0(0)	2(1.09)	1(0.68)	8(5.16)	8(4.04)	20(11.11)	39(4.29)
	44(100)	184(100.01)	148(100)	155(100.01)	198(100)	180(100.01)	909(100)

Data are n(%).

MSM, Man who have sex with man.

North, Beijing, Tianjin, Hebei, Shanxi, and Inner Mongolia.

North-east, Liaoning, Jilin, and Heilongjiang.

East, Shanghai, Jiangsu, Zhejiang, Anhui, Fujian, Jiangxi, and Shandong.

Central- south, Henan, Hubei, Hunan, Guangdong, Guangxi, and Hainan.

South-west, Chongqing, Sichuan, Guizhou, Yunnan, and Tibet.

North-west, Shann'xi, Gansu, Qinghai, Ningxia, and Sinkiang.

URF, unique recombinant form.

Minor,A1, D, F1, G, H, CRF02_AG, CRF03_AB, CRF06_cpx, CRF15_01B, CRF18_cpx, CRF24_BG, CRF33_01B, CRF55_01B, CRF57_BC, CRF58_01B, CRF59_01B, CRF61_BC, CRF62_BC, CRF63_02A1, CRF64_BC, CRF65_cpx, CRF67_01B, CRF68_01B, CRF78_cpx,

CRF79_0107, CRF82_cpx, CRF83_cpx, CRF85_BC, CRF86_BC, CRF87_cpx, CRF88_BC, and CRF96_cpx.

Table S3. HIV subtype trend for sex

	1994-2005	2006-2008	2009-2011	2012-2014	2015-2017	2018-2020	Total
Men							
01_AE	25(14.79)	417(35.89)	2148(51.61)	2387(50.64)	1747(47.85)	1477(44.85)	8201(47.82)
07_BC	52(30.77)	295(25.39)	1047(25.16)	1321(28.02)	1046(28.65)	1001(30.4)	4762(27.77)
08_BC	3(1.78)	16(1.38)	16(0.38)	21(0.45)	26(0.71)	22(0.67)	104(0.61)
55_01B	0(0)	27(2.32)	98(2.35)	142(3.01)	65(1.78)	93(2.82)	425(2.48)
B	80(47.34)	368(31.67)	696(16.72)	525(11.14)	346(9.48)	202(6.13)	2217(12.93)
C	4(2.37)	13(1.12)	10(0.24)	22(0.47)	19(0.52)	11(0.33)	79(0.46)
Minor	4(2.37)	14(1.2)	62(1.49)	64(1.36)	92(2.52)	125(3.8)	361(2.1)
URF	1(0.59)	12(1.03)	85(2.04)	232(4.92)	310(8.49)	362(10.99)	1002(5.84)
	169(100.01)	1162(100)	4162(99.99)	4714(100.01)	3651(100)	3293(99.99)	17151(100.01)
Women							
01_AE	16(22.86)	25(13.3)	53(28.19)	49(28.65)	50(28.9)	91(34.08)	284(26.87)
07_BC	11(15.71)	58(30.85)	50(26.6)	48(28.07)	61(35.26)	95(35.58)	323(30.56)
08_BC	3(4.29)	14(7.45)	8(4.26)	13(7.6)	7(4.05)	10(3.75)	55(5.2)
55_01B	0(0)	0(0)	0(0)	1(0.58)	1(0.58)	6(2.25)	8(0.76)
B	35(50)	75(39.89)	57(30.32)	35(20.47)	31(17.92)	33(12.36)	266(25.17)
C	1(1.43)	4(2.13)	5(2.66)	7(4.09)	8(4.62)	8(3)	33(3.12)
Minor	2(2.86)	10(5.32)	11(5.85)	9(5.26)	9(5.2)	12(4.49)	53(5.01)
URF	2(2.86)	2(1.06)	4(2.13)	9(5.26)	6(3.47)	12(4.49)	35(3.31)
Total	70(100.01)	188(100)	188(100.01)	171(99.98)	173(100)	267(100)	1057(100)

URF, unique recombinant form.

Minor,A1, D, F1, G, H, CRF02_AG, CRF03_AB, CRF06_cpx, CRF15_01B, CRF18_cpx, CRF24_BG, CRF33_01B, CRF55_01B, CRF57_BC, CRF58_01B, CRF59_01B, CRF61_BC, CRF62_BC, CRF63_02A1, CRF64_BC, CRF65_cpx, CRF67_01B, CRF68_01B, CRF78_cpx, CRF79_0107, CRF82_cpx, CRF83_cpx, CRF85_BC, CRF86_BC, CRF87_cpx, CRF88_BC, and CRF96_cpx.

Table S4. HIV subtype trend for ethnicity

	1994-2005	2006-2008	2009-2011	2012-2014	2015-2017	2018-2020	Total
Han							
01_AE	29(16.76)	262(34.66)	869(48.55)	1326(52.93)	1728(47.37)	1520(44.16)	5734(46.56)
07_BC	20(11.56)	108(14.29)	360(20.11)	577(23.03)	1025(28.1)	1045(30.36)	3135(25.46)
08_BC	5(2.89)	22(2.91)	16(0.89)	25(1)	31(0.85)	31(0.9)	130(1.06)
55_01B	0(0)	2(0.26)	12(0.67)	23(0.92)	64(1.75)	95(2.76)	196(1.59)
B	107(61.85)	329(43.52)	447(24.97)	373(14.89)	368(10.09)	230(6.68)	1854(15.06)
C	3(1.73)	10(1.32)	12(0.67)	19(0.76)	26(0.71)	19(0.55)	89(0.72)
Minor	6(3.47)	17(2.25)	28(1.56)	34(1.36)	99(2.71)	136(3.95)	320(2.6)
URF	3(1.73)	6(0.79)	46(2.57)	128(5.11)	307(8.42)	366(10.63)	856(6.95)
Total	173(99.99)	756(100)	1790(99.99)	2505(100)	3648(100)	3442(99.99)	12314(100)
Uyghur							
01_AE	0(0)	2(1.44)	1(1.85)	1(3.45)	0(0)	1(16.67)	5(1.69)
07_BC	40(100)	130(93.53)	50(92.59)	26(89.66)	26(96.3)	5(83.33)	277(93.9)
08_BC	0(0)	0(0)	0(0)	0(0)	0(0)	0(0)	0(0)
55_01B	0(0)	0(0)	0(0)	0(0)	0(0)	0(0)	0(0)
B	0(0)	3(2.16)	3(5.56)	2(6.9)	0(0)	0(0)	8(2.71)
C	0(0)	1(0.72)	0(0)	0(0)	0(0)	0(0)	1(0.34)
Minor	0(0)	1(0.72)	0(0)	0(0)	1(3.7)	0(0)	2(0.68)
URF	0(0)	2(1.44)	0(0)	0(0)	0(0)	0(0)	2(0.68)
Total	40(100)	139(100.01)	54(100)	29(100.01)	27(100)	6(100)	295(100)
Yi							
01_AE	0(0)	1(2.86)	0(0)	4(11.76)	0(0)	0(0)	5(3.55)
07_BC	1(50)	33(94.29)	32(84.21)	28(82.35)	17(85)	10(83.33)	121(85.82)
08_BC	0(0)	0(0)	1(2.63)	1(2.94)	2(10)	0(0)	4(2.84)
55_01B	0(0)	0(0)	0(0)	0(0)	0(0)	0(0)	0(0)
B	0(0)	0(0)	3(7.89)	0(0)	0(0)	0(0)	3(2.13)
C	1(50)	1(2.86)	0(0)	0(0)	0(0)	0(0)	2(1.42)
Minor	0(0)	0(0)	0(0)	0(0)	0(0)	0(0)	0(0)
URF	0(0)	0(0)	2(5.26)	1(2.94)	1(5)	2(16.67)	6(4.26)
Total	2(100)	35(100.01)	38(99.99)	34(99.99)	20(100)	12(100)	141(100.02)
Other ethnicities							
01_AE	4(50)	12(18.75)	32(42.11)	56(50.91)	67(54.92)	47(47)	218(45.42)
07_BC	0(0)	14(21.88)	18(23.68)	27(24.55)	37(30.33)	36(36)	132(27.5)
08_BC	0(0)	6(9.38)	3(3.95)	3(2.73)	0(0)	1(1)	13(2.71)
55_01B	0(0)	0(0)	1(1.32)	1(0.91)	2(1.64)	4(4)	8(1.67)
B	3(37.5)	26(40.63)	19(25)	11(10)	9(7.38)	5(5)	73(15.21)
C	1(12.5)	3(4.69)	0(0)	2(1.82)	1(0.82)	0(0)	7(1.46)
Minor	0(0)	2(3.13)	2(2.63)	2(1.82)	1(0.82)	1(1)	8(1.67)
URF	0(0)	1(1.56)	1(1.32)	8(7.27)	5(4.1)	6(6)	21(4.38)
Total	8(100)	64(100.02)	76(100.01)	110(100.01)	122(100.01)	100(100)	480(100.02)

Data are n(%).

URF, unique recombinant form.

Minor,A1, D, F1, G, H, CRF02_AG, CRF03_AB, CRF06_cpx, CRF15_01B, CRF18_cpx, CRF24_BG, CRF33_01B, CRF55_01B, CRF57_BC, CRF58_01B, CRF59_01B, CRF61_BC, CRF62_BC, CRF63_02A1, CRF64_BC, CRF65_cpx, CRF67_01B, CRF68_01B, CRF78_cpx, CRF79_0107, CRF82_cpx, CRF83_cpx, CRF85_BC, CRF86_BC, CRF87_cpx, CRF88_BC, and CRF96_cpx.

Table S5. HIV subtype trend for age (years)

	1994-2005	2006-2008	2009-2011	2012-2014	2015-2017	2018-2020	Total
<18							
01_AE	2(13.33)	2(7.41)	3(20)	4(50)	4(25)	5(55.56)	20(22.22)
07_BC	5(33.33)	6(22.22)	4(26.67)	1(12.5)	8(50)	2(22.22)	26(28.89)
08_BC	0(0)	1(3.7)	0(0)	0(0)	0(0)	0(0)	1(1.11)
55_01B	0(0)	0(0)	0(0)	0(0)	1(6.25)	0(0)	1(1.11)
B	8(53.33)	15(55.56)	6(40)	2(25)	1(6.25)	1(11.11)	33(36.67)
C	0(0)	1(3.7)	0(0)	1(12.5)	1(6.25)	0(0)	3(3.33)
Minor	0(0)	2(7.41)	1(6.67)	0(0)	0(0)	0(0)	3(3.33)
URF	0(0)	0(0)	1(6.67)	0(0)	1(6.25)	1(11.11)	3(3.33)
Total	15(99.99)	27(100)	15(100.01)	8(100)	16(100)	9(100)	90(99.99)
18-24							
01_AE	9(24.32)	62(31.79)	194(55.27)	210(53.85)	304(48.87)	208(42.19)	987(47.27)
07_BC	11(29.73)	66(33.85)	82(23.36)	106(27.18)	210(33.76)	186(37.73)	661(31.66)
08_BC	0(0)	7(3.59)	2(0.57)	0(0)	2(0.32)	1(0.2)	12(0.57)
55_01B	0(0)	0(0)	3(0.85)	7(1.79)	10(1.61)	11(2.23)	31(1.48)
B	14(37.84)	54(27.69)	54(15.38)	38(9.74)	42(6.75)	20(4.06)	222(10.63)
C	0(0)	3(1.54)	1(0.28)	1(0.26)	1(0.16)	4(0.81)	10(0.48)
Minor	1(2.7)	2(1.03)	7(1.99)	6(1.54)	8(1.29)	16(3.25)	40(1.92)
URF	2(5.41)	1(0.51)	8(2.28)	22(5.64)	45(7.23)	47(9.53)	125(5.99)
Total	37(100)	195(100)	351(99.98)	390(100)	622(99.99)	493(100)	2088(100)
25-44							
01_AE	15(10.2)	183(28.33)	584(45.38)	978(53.65)	1113(49.42)	993(46.34)	3866(46.59)
07_BC	45(30.61)	188(29.1)	303(23.54)	432(23.7)	623(27.66)	634(29.58)	2225(26.81)
08_BC	5(3.4)	18(2.79)	9(0.7)	21(1.15)	18(0.8)	16(0.75)	87(1.05)
55_01B	0(0)	2(0.31)	7(0.54)	14(0.77)	37(1.64)	57(2.66)	117(1.41)
B	72(48.98)	228(35.29)	324(25.17)	260(14.26)	230(10.21)	132(6.16)	1246(15.02)
C	5(3.4)	8(1.24)	9(0.7)	10(0.55)	8(0.36)	10(0.47)	50(0.6)
Minor	5(3.4)	13(2.01)	18(1.4)	20(1.1)	42(1.87)	69(3.22)	167(2.01)
URF	0(0)	6(0.93)	33(2.56)	88(4.83)	181(8.04)	232(10.83)	540(6.51)
Total	147(99.99)	646(100)	1287(99.99)	1823(100.01)	2252(100)	2143(100.01)	8298(100)
45-64							
01_AE	6(28.57)	26(23.21)	114(41.76)	171(42.75)	338(41.17)	316(40.46)	971(40.32)
07_BC	0(0)	21(18.75)	62(22.71)	104(26)	221(26.92)	227(29.07)	635(26.37)
08_BC	0(0)	2(1.79)	7(2.56)	7(1.75)	11(1.34)	9(1.15)	36(1.5)
55_01B	0(0)	0(0)	3(1.1)	3(0.75)	17(2.07)	27(3.46)	50(2.08)
B	14(66.67)	56(50)	76(27.84)	79(19.75)	93(11.33)	73(9.35)	391(16.24)
C	0(0)	3(2.68)	2(0.73)	7(1.75)	15(1.83)	4(0.51)	31(1.29)
Minor	0(0)	2(1.79)	3(1.1)	9(2.25)	47(5.72)	46(5.89)	107(4.44)
URF	1(4.76)	2(1.79)	6(2.2)	20(5)	79(9.62)	79(10.12)	187(7.77)
Total	21(100)	112(100.01)	273(100)	400(100)	821(100)	781(100.01)	2408(100.01)
≥65							
01_AE	1(33.33)	4(40)	6(20)	24(42.11)	35(33.33)	41(32.28)	111(33.43)

07_BC	0(0)	1(10)	9(30)	15(26.32)	43(40.95)	46(36.22)	114(34.34)
08_BC	0(0)	0(0)	2(6.67)	1(1.75)	2(1.9)	6(4.72)	11(3.31)
55_01B	0(0)	0(0)	0(0)	0(0)	1(0.95)	4(3.15)	5(1.51)
B	2(66.67)	4(40)	11(36.67)	7(12.28)	11(10.48)	9(7.09)	44(13.25)
C	0(0)	0(0)	0(0)	2(3.51)	2(1.9)	1(0.79)	5(1.51)
Minor	0(0)	1(10)	1(3.33)	1(1.75)	4(3.81)	6(4.72)	13(3.92)
URF	0(0)	0(0)	1(3.33)	7(12.28)	7(6.67)	14(11.02)	29(8.73)
Total	3(100)	10(100)	30(100)	57(100)	105(99.99)	127(99.99)	332(100)

Data are n(%).

URF, unique recombinant form.

Minor, A1, D, F1, G, H, CRF02_AG, CRF03_AB, CRF06_cpx, CRF15_01B, CRF18_cpx, CRF24_BG, CRF33_01B, CRF55_01B, CRF57_BC, CRF58_01B, CRF59_01B, CRF61_BC, CRF62_BC, CRF63_02A1, CRF64_BC, CRF65_cpx, CRF67_01B, CRF68_01B, CRF78_cpx, CRF79_0107, CRF82_cpx, CRF83_cpx, CRF85_BC, CRF86_BC, CRF87_cpx, CRF88_BC, and CRF96_cpx.

Table S6. Characteristics of clusters for subtype B, CRF01_AE, and CRF07_BC

	Men	Women	CD4	Age	Han	Uyghur	Yi	Others	Heterosexual	MSM	IDU	Blood transmission	Mother to child	North	North-east	East	Central-south	South-west	North-west
B Cluster																			
1	1769(97.36)	48(2.64)	306(178-450)	32(27-40)	1291(95.35)	4(0.3)	3(0.22)	56(4.14)	363(19.61)	1446(78.12)	21(1.13)	21(1.13)	0(0)	954(51.21)	214(11.49)	292(15.67)	252(13.53)	82(4.4)	69(3.7)
2	111(63.43)	64(36.57)	258(92-375.5)	39(33-51)	159(96.36)	1(0.61)	0(0)	5(3.03)	134(44.67)	28(9.33)	34(11.33)	101(33.67)	3(1)	63(20.66)	25(8.2)	47(15.41)	114(37.38)	50(16.39)	6(1.97)
3	286(70.44)	120(29.56)	315(174-428)	38(29-49)	326(96.45)	1(0.3)	0(0)	11(3.25)	194(34.22)	154(27.16)	7(1.23)	205(36.16)	7(1.23)	159(27.65)	34(5.91)	94(16.35)	259(45.04)	14(2.43)	15(2.61)
01AE Cluster																			
1	1881(97.41)	50(2.59)	335(204-466)	31(26-41)	1345(95.46)	0(0)	2(0.14)	62(4.4)	356(18.28)	1571(80.69)	19(0.98)	1(0.05)	0(0)	793(40.63)	541(27.72)	247(12.65)	262(13.42)	58(2.97)	51(2.61)
2	383(79.13)	101(20.87)	285(93-410)	37(28-49.75)	366(97.86)	0(0)	0(0)	8(2.14)	592(64.77)	224(24.51)	72(7.88)	20(2.19)	6(0.66)	166(18.06)	38(4.13)	192(20.89)	410(44.61)	107(11.64)	6(0.65)
3	392(98.99)	4(1.01)	344(235-484)	30(26-38.5)	308(96.55)	0(0)	0(0)	11(3.45)	70(17.68)	324(81.82)	2(0.51)	0(0)	0(0)	206(52.02)	41(10.35)	74(18.69)	43(10.86)	19(4.8)	13(3.28)
4	1360(97.98)	28(2.02)	337(215-461)	30(25-37)	969(96.51)	1(0.1)	1(0.1)	33(3.29)	237(16.64)	1164(81.74)	18(1.26)	5(0.35)	0(0)	638(44.87)	148(10.41)	293(20.6)	238(16.74)	53(3.73)	52(3.66)
5	2352(98.7)	31(1.3)	321(178-456)	30(26-37)	1245(95.92)	2(0.15)	0(0)	51(3.93)	349(14.2)	2080(84.62)	19(0.77)	10(0.41)	0(0)	676(27.48)	207(8.41)	996(40.49)	370(15.04)	134(5.45)	77(3.13)
6	232(99.57)	1(0.43)	351(219-500)	33(28-46.25)	181(94.27)	0(0)	0(0)	11(5.73)	34(14.47)	199(84.68)	2(0.85)	0(0)	0(0)	144(61.28)	22(9.36)	25(10.64)	28(11.91)	7(2.98)	9(3.83)
7	295(96.72)	10(3.28)	316(123-422.25)	33(27-44)	251(97.67)	0(0)	0(0)	6(2.33)	66(22)	228(76)	5(1.67)	1(0.33)	0(0)	151(50)	41(13.58)	40(13.25)	41(13.58)	10(3.31)	19(6.29)
07BC Cluster																			
1	2888(97.44)	76(2.56)	363(255-500)	31(26-42)	1913(96.57)	0(0)	3(0.15)	65(3.28)	476(15.9)	2485(83)	27(0.9)	6(0.2)	0(0)	1023(34.09)	281(9.36)	589(19.63)	681(22.69)	324(10.8)	103(3.43)
2	759(77.13)	225(22.87)	364(241-506)	33(26-41)	482(52.97)	276(30.33)	117(12.86)	35(3.85)	430(25.97)	223(13.47)	979(59.12)	21(1.27)	3(0.18)	277(16.61)	72(4.32)	98(5.88)	181(10.85)	709(42.51)	331(19.84)
3	825(97.75)	19(2.25)	362(257-487)	31(26-41)	540(96.09)	1(0.18)	1(0.18)	20(3.56)	133(15.38)	718(83.01)	11(1.27)	3(0.35)	0(0)	310(35.8)	77(8.89)	161(18.59)	217(25.06)	75(8.66)	26(3)
4	259(98.85)	3(1.15)	417(275-515)	30(26-37)	177(94.15)	0(0)	0(0)	11(5.85)	47(17.67)	217(81.58)	1(0.38)	1(0.38)	0(0)	113(42.16)	29(10.82)	32(11.94)	69(25.75)	15(5.6)	10(3.73)

Data are n(%), or median (IQR).

MSM, Man who have sex with man.

North, Beijing, Tianjin, Hebei, Shanxi, and Inner Mongolia.

North-east, Liaoning, Jilin, and Heilongjiang.

East, Shanghai, Jiangsu, Zhejiang, Anhui, Fujian, Jiangxi, and Shandong.

Central- south, Henan, Hubei, Hunan, Guangdong, Guangxi, and Hainan.

South-west, Chongqing, Sichuan, Guizhou, Yunnan, and Tibet.

North-west, Shann'xi, Gansu, Qinghai, Ningxia, and Sinkiang.

Table S7. Risk factors for slower progression to AIDSAD in Chinese patients (excluding heterosexual)

	Number of sequences	AIDSAD prevalence[a]	Univariable analysis[b]		Multivariable analysis[c]	
			OR (95% CI)	P value	OR (95% CI)	P value
Sex						
Men	7483	1543(20.62)	Reference			
Women	99	36(36.36)	2.2(1.44-3.31)	0.001	1.07(0.58-1.94)	0.81
Age at diagnosis(years) group						
<18	43	8(18.6)	Reference			
18-24	1331	166(12.47)	0.62(0.3-1.47)	0.24	1.56(0.6-4.98)	0.4
25-44	4985	1027(20.6)	1.14(0.55-2.64)	0.75	2.75(1.07-8.69)	0.05
45-64	1109	341(30.75)	1.94(0.94-4.55)	0.09	4.6(1.78-14.57)	0.001
≥65	111	36(32.43)	2.1(0.92-5.28)	0.09	5.88(2.09-19.74)	0.001
Ethnicity						
Han	7173	1508(21.02)	Reference			
Uyghur	79	8(10.13)	0.42(0.19-0.83)	0.02	0.74(0.3-1.6)	0.47
Yi	72	7(9.72)	0.4(0.17-0.82)	0.02	0.63(0.25-1.39)	0.28
Other	258	56(21.71)	1.04(0.76-1.4)	0.79	1.11(0.81-1.5)	0.51
Transmission risk group						
MSM	7084	1454(20.53)	Reference			
Injecting drug user	312	51(16.35)	0.76(0.55-1.02)	0.07	1.16(0.78-1.67)	0.45
Blood transfusion	112	51(45.54)	3.24(2.22-4.71)	0	2.72(1.64-4.51)	0.001
Mother to child	10	4(40)	2.58(0.66-9.05)	0.14	6.4(1.22-33.1)	0.02
Subtype						
B	1009	283(28.05)	Reference			
C	25	5(20)	0.64(0.21-1.6)	0.38	0.74(0.24-1.94)	0.57
01_AE	3663	855(23.34)	0.78(0.67-0.92)	0	0.85(0.72-1.01)	0.06
07_BC	2069	281(13.58)	0.4(0.33-0.49)	0.0001	0.42(0.34-0.51)	0.0001

08_BC	27	10(37.04)	1.51(0.66-3.28)	0.31	1.42(0.61-3.16)	0.4
55_01B	122	22(18.03)	0.56(0.34-0.9)	0.02	0.53(0.31-0.85)	0.01
URF	526	93(17.68)	0.55(0.42-0.71)	0	0.53(0.4-0.7)	0.001
Minor	1009	283(28.05)	0.69(0.45-1.05)	0.09	0.6(0.38-0.92)	0.02
Region						
North	3606	758(21.02)	Reference			
North-east	1138	235(20.65)	0.98(0.83-1.15)	0.79		
East	941	192(20.4)	0.96(0.8-1.15)	0.68		
Central-south	955	207(21.68)	1.04(0.87-1.23)	0.66		
South-west	515	108(20.97)	1(0.79-1.25)	0.98		
North-west	404	72(17.82)	0.81(0.62-1.06)	0.13		
Sampling Phase						
1994-2005	48	16(33.33)	Reference			
2006-2008	393	85(21.63)	0.55(0.29-1.08)	0.07	0.66(0.33-1.36)	0.25
2009-2011	1126	232(20.6)	0.52(0.28-0.98)	0.04	0.74(0.38-1.51)	0.4
2012-2014	1971	362(18.37)	0.45(0.25-0.85)	0.01	0.71(0.36-1.44)	0.32
2015-2017	2429	531(21.86)	0.56(0.31-1.05)	0.06	0.92(0.47-1.87)	0.81
2018-2020	1615	353(21.86)	0.56(0.31-1.06)	0.06	0.94(0.48-1.92)	0.85

AIDSAD, AIDS at diagnose.

OR, odds ratio.

MSM, Man who have sex with man.

URF, unique recombinant form.

North, Beijing, Tianjin, Hebei, Shanxi, and Inner Mongolia.

North-east, Liaoning, Jilin, and Heilongjiang.

East, Shanghai, Jiangsu, Zhejiang, Anhui, Fujian, Jiangxi, and Shandong.

Central- south, Henan, Hubei, Hunan, Guangdong, Guangxi, and Hainan.

South-west, Chongqing, Sichuan, Guizhou, Yunnan, and Tibet.

North-west, Shann'xi, Gansu, Qinghai, Ningxia, and Sinkiang.

Minor,A1, D, F1, G, H, CRF02_AG, CRF03_AB, CRF06_cpx, CRF15_01B, CRF18_cpx, CRF24_BG, CRF33_01B, CRF55_01B, CRF57_BC, CRF58_01B, CRF59_01B, CRF61_BC, CRF62_BC, CRF63_02A1, CRF64_BC, CRF65_cpx, CRF67_01B, CRF68_01B, CRF78_cpx, CRF79_0107, CRF82_cpx, CRF83_cpx, CRF85_BC, CRF86_BC, CRF87_cpx, CRF88_BC, and CRF96_cpx.

aData are n (%).

bUnivariable logistic regression analysis.

cMultivariable logistic regression analysis.

Table S8. Risk factors for slower progression to AIDSAD in Chinese patients(excluding MSM)

	Number of sequences	AIDSAD prevalence[a]	Univariable analysis[b]		Multivariable analysis[c]	
			OR (95% CI)	P value	OR (95% CI)	P value
Sex						
Men	2597	756(29.11)	Reference			
Women	758	210(27.7)	0.93(0.78-1.12)	0.45	0.88(0.72-1.07)	0.19
Age at diagnosis(years) group						
<18	26	9(34.62)	Reference			
18-24	354	45(12.71)	0.28(0.12-0.68)	0.004	0.56(0.18-1.86)	0.32
25-44	1909	516(27.03)	0.7(0.32-1.65)	0.39	1.28(0.44-4.1)	0.66
45-64	927	337(36.35)	1.08(0.49-2.56)	0.86	1.96(0.67-6.3)	0.23
≥65	136	58(42.65)	1.4(0.6-3.51)	0.45	2.99(0.97-10.03)	0.06
Ethnicity						
Han	91	8(8.79)	Reference			
Uyghur	81	5(6.17)	0.23(0.1-0.44)	0.0001	0.45(0.18-1.03)	0.07
Yi	131	44(33.59)	0.16(0.05-0.35)	0.0001	0.32(0.11-0.79)	0.02
Other	91	8(8.79)	1.19(0.82-1.72)	0.35	1.34(0.89-1.98)	0.15
Transmission risk group						
Heterosexual	2857	841(29.44)	Reference			
Injecting drug user	312	51(16.35)	0.47(0.34-0.63)	0.0001	0.87(0.6-1.24)	0.45
Blood transfusion	112	51(45.54)	2(1.37-2.93)	0.0001	1.84(1.2-2.82)	0.0001
Mother to child	10	4(40)	1.6(0.41-5.61)	0.47	3.23(0.55-18.82)	0.18
Subtype						
B	625	228(36.48)	Reference			
C	53	16(30.19)	0.75(0.4-1.36)	0.36	0.88(0.46-1.64)	0.7
01_AE	1256	430(34.24)	0.91(0.74-1.11)	0.34	0.98(0.79-1.23)	0.89
07_BC	974	172(17.66)	0.37(0.3-0.47)	0.0001	0.44(0.34-0.57)	0.0001
08_BC	77	22(28.57)	0.7(0.41-1.16)	0.17	0.67(0.38-1.14)	0.15

55_01B	49	19(38.78)	1.1(0.6-1.99)	0.75	1.16(0.62-2.13)	0.63
URF	201	52(25.87)	0.61(0.42-0.86)	0.01	0.64(0.44-0.93)	0.02
Minor	625	228(36.48)	0.51(0.31-0.79)	0.004	0.54(0.33-0.86)	0.01
Region						
North	1594	468(29.36)	Reference			
North-east	389	113(29.05)	0.99(0.77-1.25)	0.9	1.01(0.78-1.3)	0.95
East	340	108(31.76)	1.12(0.87-1.44)	0.38	1.1(0.84-1.43)	0.5
Central-south	457	147(32.17)	1.14(0.91-1.43)	0.25	1.01(0.79-1.29)	0.94
South-west	338	75(22.19)	0.69(0.52-0.9)	0.01	1.14(0.83-1.55)	0.42
North-west	219	48(21.92)	0.68(0.48-0.94)	0.02	1.26(0.84-1.86)	0.01
Sampling Phase						
1994-2005	52	16(30.77)	Reference			
2006-2008	315	86(27.3)	0.84(0.45-1.64)	0.61		
2009-2011	527	179(33.97)	1.16(0.64-2.2)	0.64		
2012-2014	715	173(24.2)	0.72(0.4-1.36)	0.29		
2015-2017	742	218(29.38)	0.94(0.52-1.77)	0.83		
2018-2020	1004	294(29.28)	0.93(0.52-1.75)	0.82		

AIDSAD, AIDS at diagnose.

OR, odds ratio.

MSM, Man who have sex with man.

URF, unique recombinant form.

North, Beijing, Tianjin, Hebei, Shanxi, and Inner Mongolia.

North-east, Liaoning, Jilin, and Heilongjiang.

East, Shanghai, Jiangsu, Zhejiang, Anhui, Fujian, Jiangxi, and Shandong.

Central- south, Henan, Hubei, Hunan, Guangdong, Guangxi, and Hainan.

South-west, Chongqing, Sichuan, Guizhou, Yunnan, and Tibet.

North-west, Shann'xi, Gansu, Qinghai, Ningxia, and Sinkiang.

Minor,A1, D, F1, G, H, CRF02_AG, CRF03_AB, CRF06_cpx, CRF15_01B, CRF18_cpx, CRF24_BG, CRF33_01B, CRF55_01B, CRF57_BC, CRF58_01B, CRF59_01B, CRF61_BC, CRF62_BC, CRF63_02A1, CRF64_BC, CRF65_cpx, CRF67_01B, CRF68_01B, CRF78_cpx, CRF79_0107, CRF82_cpx, CRF83_cpx, CRF85_BC, CRF86_BC, CRF87_cpx, CRF88_BC, and CRF96_cpx.

aData are n (%).

bUnivariable logistic regression analysis.

cMultivariable logistic regression analysis.

Table S9. Risk factors for slower progression to AIDSAD in Chinese patients (excluding injecting drug user)

	Number of sequences	AIDSAD prevalence[a]	Univariable analysis[b]		Multivariable analysis[c]	
			OR (95% CI)	P value	OR (95% CI)	P value
Sex						
Men	9403	2166(23.04)	Reference			
Women	724	203(28.04)	1.3(1.1-1.54)	0.002	0.85(0.7-1.04)	0.11
Age at diagnosis(years) group						
<18	48	10(20.83)	Reference			
18-24	1577	205(13)	0.57(0.29-1.22)	0.12	1.17(0.5-3.15)	0.74
25-44	6321	1421(22.48)	1.1(0.57-2.34)	0.79	2.1(0.91-5.62)	0.11
45-64	1937	642(33.14)	1.88(0.97-4.02)	0.08	3.49(1.51-9.39)	0.01
≥65	240	91(37.92)	2.32(1.14-5.13)	0.03	4.82(2-13.33)	0.001
Ethnicity						
Han	9711	2274(23.42)	Reference			
Uyghur	26	1(3.85)	0.13(0.01-0.62)	0.05	0.22(0.01-1.09)	0.15
Yi	27	2(7.41)	0.26(0.04-0.88)	0.07	0.37(0.06-1.28)	0.18
Other	363	92(25.34)	1.11(0.87-1.41)	0.4	1.14(0.89-1.46)	0.3
Transmission risk group						
Heterosexual	2857	841(29.44)	Reference			
MSM	7084	1454(20.53)	0.62(0.56-0.68)	0.001	0.67(0.6-0.75)	0.001
Blood transfusion	112	51(45.54)	2(1.37-2.93)	0.001	1.9(1.26-2.86)	0.001
Mother to child	10	4(40)	1.6(0.41-5.61)	0.47	3.68(0.74-17.54)	0.1
Subtype						
B	1488	444(29.84)	Reference			
C	70	19(27.14)	0.88(0.5-1.48)	0.63	0.8(0.45-1.37)	0.42
01_AE	4753	1246(26.22)	0.84(0.74-0.95)	0.01	0.95(0.83-1.09)	0.5
07_BC	2619	397(15.16)	0.42(0.36-0.49)	0.0001	0.46(0.39-0.54)	0.0001

08_BC	87	26(29.89)	1(0.62-1.59)	0.99	0.79(0.48-1.27)	0.34
55_01B	167	41(24.55)	0.77(0.52-1.1)	0.16	0.8(0.54-1.16)	0.25
URF	693	140(20.2)	0.6(0.48-0.74)	0.001	0.63(0.51-0.79)	0.001
Minor	1488	444(29.84)	0.68(0.49-0.93)	0.02	0.59(0.42-0.82)	0.002
Region						
North	4987	1178(23.62)	Reference			
North-east	1459	334(22.89)	0.96(0.83-1.1)	0.56	0.96(0.83-1.1)	0.56
East	1237	291(23.52)	0.99(0.86-1.15)	0.94	0.99(0.86-1.15)	0.94
Central-south	1298	304(23.42)	0.99(0.86-1.14)	0.88	0.99(0.86-1.14)	0.88
South-west	658	154(23.4)	0.99(0.81-1.19)	0.9	0.99(0.81-1.19)	0.9
North-west	465	101(21.72)	0.9(0.71-1.12)	0.36	0.9(0.71-1.12)	0.36
Sampling Phase						
1994-2005	64	18(28.13)	Reference			
2006-2008	518	123(23.75)	0.8(0.45-1.45)	0.44	0.8(0.45-1.45)	0.44
2009-2011	1476	360(24.39)	0.82(0.48-1.48)	0.5	0.82(0.48-1.48)	0.5
2012-2014	2461	503(20.44)	0.66(0.38-1.17)	0.14	0.66(0.38-1.17)	0.14
2015-2017	3039	724(23.82)	0.8(0.47-1.42)	0.43	0.8(0.47-1.42)	0.43
2018-2020	2569	641(24.95)	0.85(0.5-1.51)	0.56	0.85(0.5-1.51)	0.56

AIDSAD, AIDS at diagnose.

OR, odds ratio.

MSM, Man who have sex with man.

URF, unique recombinant form.

North, Beijing, Tianjin, Hebei, Shanxi, and Inner Mongolia.

North-east, Liaoning, Jilin, and Heilongjiang.

East, Shanghai, Jiangsu, Zhejiang, Anhui, Fujian, Jiangxi, and Shandong.

Central- south, Henan, Hubei, Hunan, Guangdong, Guangxi, and Hainan.

South-west, Chongqing, Sichuan, Guizhou, Yunnan, and Tibet.

North-west, Shann'xi, Gansu, Qinghai, Ningxia, and Sinkiang.

Minor,A1, D, F1, G, H, CRF02_AG, CRF03_AB, CRF06_cpx, CRF15_01B, CRF18_cpx, CRF24_BG, CRF33_01B, CRF55_01B, CRF57_BC, CRF58_01B, CRF59_01B, CRF61_BC, CRF62_BC, CRF63_02A1, CRF64_BC, CRF65_cpx, CRF67_01B, CRF68_01B, CRF78_cpx, CRF79_0107, CRF82_cpx, CRF83_cpx, CRF85_BC, CRF86_BC, CRF87_cpx, CRF88_BC, and CRF96_cpx.

aData are n (%).

bUnivariable logistic regression analysis.

cMultivariable logistic regression analysis.

Table S10. Results from the multi-variable linear regression analysis

	Estimate Coefficients(IC 95%)	Std.Error	T value	P
Intercept	436.6(398.4-474.9)	19.5	22.36	<0.0001
Sex	9.2(-7.1-25.6)	8.4	1.1	0.27
Age	-42.5(-48.7--36.2)	3.2	-13.4	<0.0001
Ethnicity	0.9(-6-7.8)	3.5	0.27	0.79
Risk	14.4(6.7-22.1)	3.9	3.66	0.0002
Subtype	12.4(9.7-15)	1.3	9.2	<0.0001
Region	-4.1(-6.6--1.5)	1.3	-3.07	0.002
Period	-5.6(-9.1--2)	1.8	-3.05	0.002

Sex:Man,1;Woman,2

Age(years):<18 ,1; (18-24) ,2;(25-44) ,3; (45-64),4; >65,5

Transmission risk group:Heterosexual,1; MSM,2; IDU,3; Blood,4; Mother to child,5

Subtype: B,1; C,2; 01_AE,3; 07_BC,4; 08_BC,5, 55_01B,6; URF,7; Other, 8

Ethnicity: Han, 1; Uygur,2; Yi,3; Other,4

Region: North,1; North-east,2; East,3; Central-south,4; South-west,5; North-west,6

Period (Calender year): 1994-2005,1; 2006-2008,2; 2009-2011,3; 2012-2014,4; 2015-2017,5; 2018-2020,6

Table S11. Correlation matrix of the variables

	CD4	Sex	Age	Ethnicity	Risk	Subtype	Region	Period
CD4	1	-0.01	-0.14	0.01	0.06	0.08	-0.01	-0.03
Sex	-0.01	1	0.08	0.03	-0.24	-0.03	0.06	-0.08
Age	-0.14	0.08	1	-0.03	-0.18	0.04	-0.13	0.11
Ethnicity	0.01	0.03	-0.03	1	0.04	0	0.08	-0.05
Risk	0.06	-0.24	-0.18	0.04	1	-0.03	0.08	-0.15
Subtype	0.08	-0.03	0.04	0	-0.03	1	0.02	0.26
Region	-0.01	0.06	-0.13	0.08	0.08	0.02	1	-0.05
Period	-0.03	-0.08	0.11	-0.05	-0.15	0.26	-0.05	1

Sex:Man,1;Woman,2

Age(years):<18 ,1; (18-24) ,2;(25-44) ,3; (45-64),4; >65,5

Transmission risk group:Heterosexual,1; MSM,2; IDU,3; Blood,4; Mother to child,5

Subtype: B,1; C,2; 01_AE,3; 07_BC,4; 08_BC,5, 55_01B,6; URF,7; Other, 8

Ethnicity: Han, 1; Uygur,2; Yi,3; Other,4

Region: North,1; North-east,2; East,3; Central-south,4; South-west,5; North-west,6

Period (Calender year): 1994-2005,1; 2006-2008,2; 2009-2011,3; 2012-2014,4; 2015-2017,5; 2018-2020,6

Table S12.Probability matrix of the variables

	CD4	Sex	Age	Ethnicity	Risk	Subtype	Region	Period
CD4	0	1	0	1	0	0	1	0.04
Sex	0.26	0	0	0.02	0	0.01	0	0
Age	0	0	0	0.01	0	0	0	0
Ethnicity	0.4	0	0	0	0	1	0	0
Risk	0	0	0	0	0	0	0	0
Subtype	0	0	0	0.94	0	0	0.09	0
Region	0.55	0	0	0	0	0.02	0	0
Period	0.01	0	0	0	0	0	0	0

Sex:Man,1;Woman,2

Age(years):<18 ,1; (18-24) ,2;(25-44) ,3; (45-64),4; >65,5

Transmission risk group:Heterosexual,1; MSM,2; IDU,3; Blood,4; Mother to child,5

Subtype: B,1; C,2; 01_AE,3; 07_BC,4; 08_BC,5, 55_01B,6; URF,7; Other, 8

Ethnicity: Han, 1; Uygur,2; Yi,3; Other,4

Region: North,1; North-east,2; East,3; Central-south,4; South-west,5; North-west,6

Period (Calender year): 1994-2005,1; 2006-2008,2; 2009-2011,3; 2012-2014,4; 2015-2017,5; 2018-2020,6
